# Muscle satellite cells and impaired late stage regeneration in different murine models for muscular dystrophies

**DOI:** 10.1038/s41598-019-48156-7

**Published:** 2019-08-14

**Authors:** Antonio F. Ribeiro, Lucas S. Souza, Camila F. Almeida, Renata Ishiba, Stephanie A. Fernandes, Danielle A. Guerrieri, André L. F. Santos, Paula C. G. Onofre-Oliveira, Mariz Vainzof

**Affiliations:** Human Genome and Stem-cell Research Center, Biosciences Institute, University of São Paulo, São Paulo, 05508-090 Brazil

**Keywords:** Mechanisms of disease, Genetics, Muscle stem cells

## Abstract

Satellite cells (SCs) are the main muscle stem cells responsible for its regenerative capacity. In muscular dystrophies, however, a failure of the regenerative process results in muscle degeneration and weakness. To analyze the effect of different degrees of muscle degeneration in SCs behavior, we studied adult muscle of the dystrophic strains: *DMD*^*mdx*^, *Large*^*myd*^, *DMD*^*mdx*^/*Large*^*myd*^, with variable histopathological alterations. Similar results were observed in the dystrophic models, which maintained normal levels of PAX7 expression, retained the Pax7-positive SCs pool, and their proliferation capacity. Moreover, elevated expression of MYOG, an important myogenic factor, was also observed. The ability to form new fibers was verified by the presence of dMyHC positive regenerating fibers. However, those fibers had incomplete maturation characteristics, such as small and homogenous fiber caliber, which could contribute to their dysfunction. We concluded that dystrophic muscles, independently of their degeneration degree, retain their SCs pool with proliferating and regenerative capacities. Nonetheless, the maturation of these new fibers is incomplete and do not prevent muscle degeneration. Taken together, these results suggest that the improvement of late muscle regeneration should better contribute to therapeutic approaches.

## Introduction

Skeletal muscle tissue represent about 40% of total body weight and is responsible for almost all voluntary movements. It has a high regenerative capacity after injury, which is directly linked to the presence of satellite cells (SCs). These cells are the main muscle stem cells and have a key role in muscle development during embryogenesis^1–3^. SCs reside between the basal lamina and the muscle membrane^4^ and although quiescent in normal adult muscles, SCs can be activated by specific signals upon muscle injury. In diseases characterized by chronic degeneration process, such as muscular dystrophies, SCs are constantly activated, leading to the depletion of the SCs pool and consequent failure of the regenerative process^5^.

Muscular dystrophies are a heterogeneous group of genetic diseases that cause a progressive loss of motor ability. Defects in components of the dystrophin-glycoprotein complex (DGC) are responsible for different forms of muscular dystrophies. The DGC links the myofiber cytoskeleton proteins and the extracellular matrix, providing mechanical support to sarcolemma during myofiber contraction^6,7^.

Mutations in the dystrophin (*DMD*) gene can lead to Duchenne/Becker muscular dystrophies^8^. DGC proteins are linked to extracellular matrix proteins by post-translational glycosylation of α-dystroglycan (α-DG) protein. Mutations in the *LARGE1* gene, which encodes a glycosyltransferase that acts in the α-DG, lead to congenital muscular dystrophy type 1D^9–11^.

Identified in nature or generated in laboratory, animal models are an important tool to study neuromuscular disorders. These models generally present physiological alterations observed in human patients and can be used for pathophysiological studies and therapy testing^12^. A good genetic and biochemical model for Duchenne muscular dystrophy is the *Dmd*^*mdx*^ mouse that bears a nonsense point mutation in exon 23 of the *Dmd* gene, which causes lack of this protein in skeletal muscle. Regardless of total protein deficiency, this mouse shows a mild phenotype, with comparatively moderate muscle pathology. Muscle histopathology analysis shows a great number of regenerating fibers, variation in fiber size and the presence of central nuclei. Differently from human Duchenne muscular dystrophy patients, muscle degeneration in *Dmd*^*mdx*^ mouse is followed by a significant regeneration^13,14^.

A model for Congenital muscular dystrophy type 1D is the myodystrophy *Large*^*myd*^ mouse, which harbors a mutation in the *Large1* gene. *Large*^*myd*^ mutant mice develop a progressive severe myopathy, with a maximum lifespan of 39 weeks. Muscle histopathology includes degeneration, loss of striation, variation in fiber size and the presence of central nuclei^15^.

With the goal of obtaining an animal model for DMD with a severe phenotype, more similar to affected DMD patients, and to better understand the interplay between the DMD protein and LARGE1 glycosyltransferase in muscle function, we generated double-mutant animals deficient in these two proteins^16^. The new *Dmd*^*mdx*^*/Large*^*myd*^ mouse model has a severe phenotype with delayed growth and development, and is unable to reproduce. In addition to the important pathophysiological aspects of these two mutations in muscle formation and function, this model has an important application for testing therapies, integrating functional, molecular and protein studies^16^.

Aiming to understand the behavior of SCs in dystrophic muscles with different degrees of muscle degeneration, we evaluated histological parameters, as well as gene and protein expression of transcription factors related to SCs, in gastrocnemius muscle of adult male mice with different forms of muscular dystrophies. The results were compared regarding regenerating and degenerating features. Our findings indicate that the pool of SCs of the studied dystrophic strains maintains its proliferative capacity and these cells can contribute to muscle regeneration and new fiber formation. Nevertheless, the maturation of these new fibers is incomplete and do not prevent muscle degeneration.

## Results

### Dystrophic muscles retain their SCs pool

**Figure 1:**
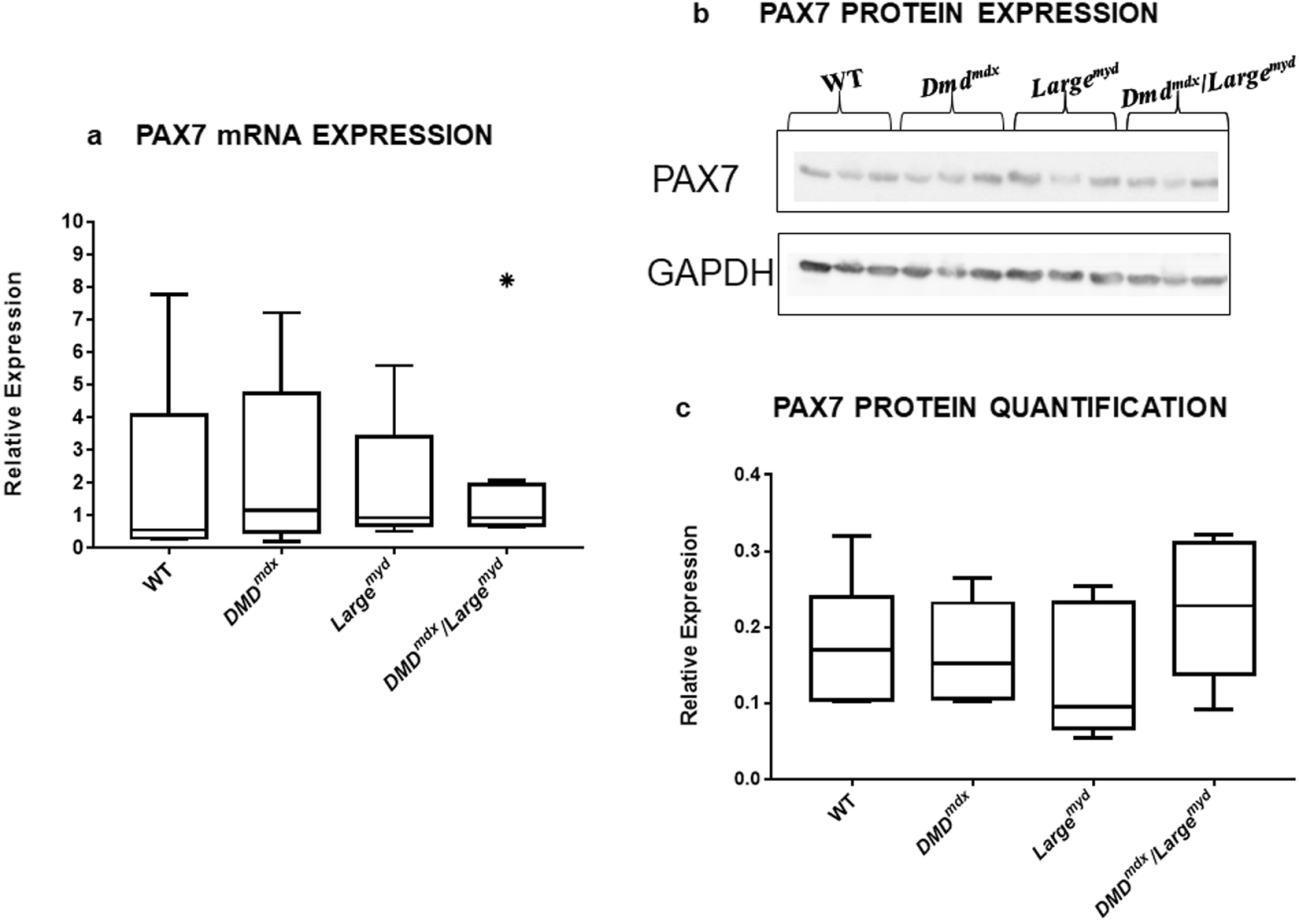
PAX7 Expression in dystrophic muscles: (**a**) Boxplot of mRNA relative Pax7 expression and (**b**) PAX7 protein western blot analysis (full blots figures without contrast adjustments are presented at Supplementary Material). N = 6 animals per strain. (**c**) Boxplot of densitometric quantification. No significant differences in PAX7 were found between WT and the three dystrophic strains using both approaches (Kruskal-Wallis test, Chi-square = 1.05, df = 3, p = 0.790). *Shows outlier measures.

The expression of the transcription factor *Pax7*, which is related to the maintenance of SCs pool, was measured to investigate a potential reduction in the SCs pool in the dystrophic mice models as compared to wild-type mice. Statistical analysis with Kruskal-Wallis test revealed similar expression of *Pax7* in the three dystrophic strains as compared to wild-type mice (*Chi-square* = 1.05, *df* = 3, *p* = 0.790), independently of the degree of muscle degeneration (Fig. 1a). Protein analysis by Western blot also showed similar expression in the dystrophic strains as compared to wild-type animals (Fig. 1b,c).

**Figure 2:**
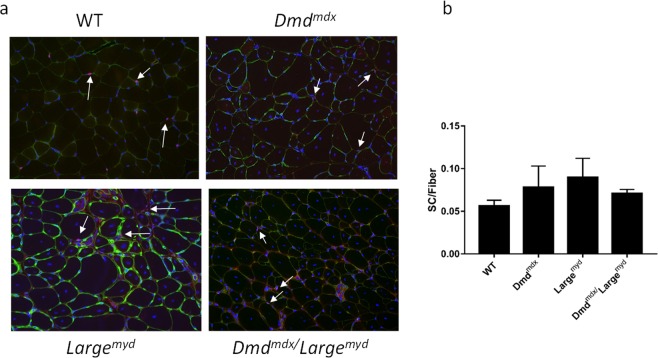
Representative images of PAX7-positive SCs in the four mouse strains, magnification 200 × (full immunofluorescence figures are represented at Supplementary Material). (**a**) Immunofluorescence analysis showing PAX7-positive and DAPI-positive cells in each strain, ~3000 fibers were counted for each animal. White arrows are indicating SCs, purple labelling. (**b**) Proportion of SCs per fibers in the four strains. No statistical significance was found among wild-type and the three dystrophic strains. N = 4 animals per strain (One-way ANOVA, F(3, 11) = 2.9, p = 0.084).

Immunofluorescence analysis using PAX7 antibody in gastrocnemius muscle sections of dystrophic versus wild-type animals showed an apparently higher number of PAX7 positive nuclei per fibers in the three dystrophic models (Fig. 2a). However, no statistical differences were found among the four groups (p = 0.083, Fig. 2b). Thus, SCs pool is maintained in spite of the different degrees of degeneration in the dystrophic mouse models.

### Number of activated satellite cells is elevated in the dystrophic mice, compatible with active regeneration and pool maintenance

**Figure 3:**
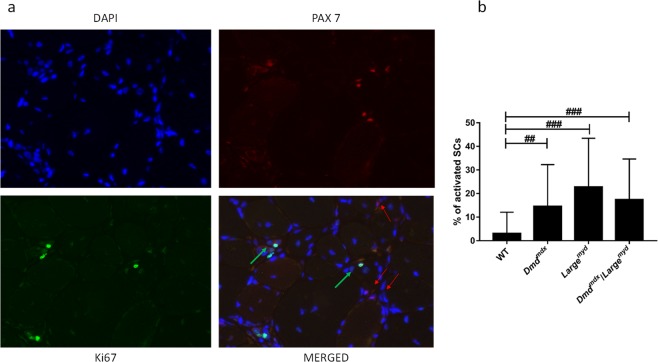
Representative images of PAX7 and KI67 positive SCs, magnification 200X (full immunofluorescence figures are represented at Supplementary Material). (**a**) Identification of quiescent and activated SCs, using as example the Large^myd^ model. On the merged figure, red arrows indicate quiescent SCs (DAPI+ and PAX7+); green arrows indicate activated SCs (DAPI+, PAX7+ and KI67+). (**b**) Percentage of activated SCs in wild-type and dystrophic models, N = 200 PAX7+ SCs per strain. Statistical difference was observed between the wild type and each one of three dystrophic models (Kruskal-Wallis test, Chi-square = 30.248, df = 3, p < 0.001, ^##^p < 0.01, ^###^p < 0.001).

Using PAX7 and KI67 antibodies, we identified and counted SCs that remained quiescent and the ones that were activated. While PAX7 staining shows SCs population, KI67 staining is a marker of proliferating cells. Therefore, the double staining for PAX7 and KI67 shows activated SCs^17^ (Fig. 3a). A higher number of activated SCs were found in the three dystrophic models, when compared with wild-type, but no differences among the dystrophic strains (Kruskal-Wallis test, *Chi-square* = 30.248, *df* = 3, *p* < 0.001). Dystrophic animals showed the following proportions of activated SCs: *DMD*^*mdx*^ 17.4%, *Large*^*myd*^ 25.1% and *DMD*^*mdx*^/*Large*^*myd*^ 16,.7% (Fig. 3b).

### Myogenic regulatory genes are activated in the dystrophic muscle

**Figure 4:**
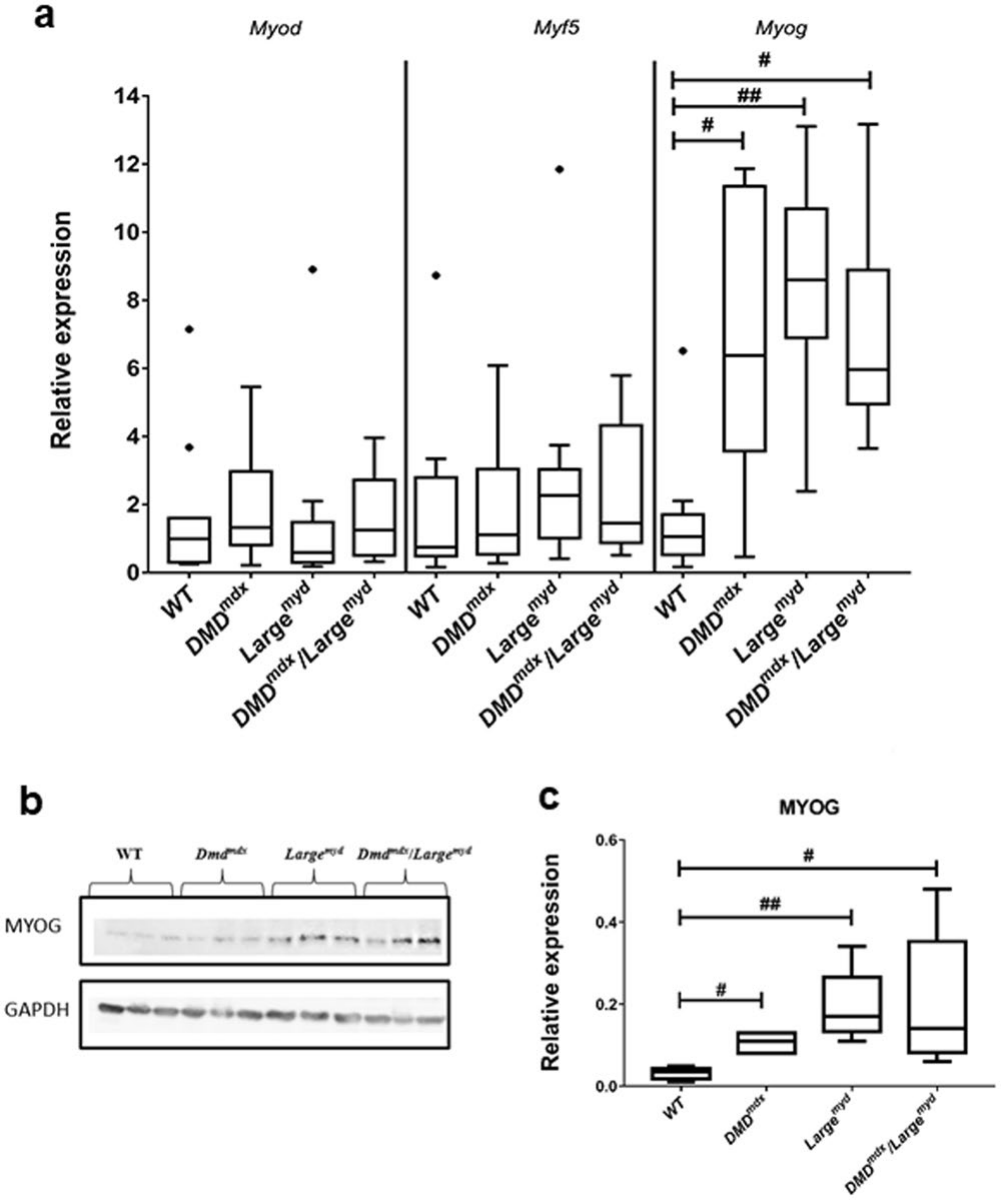
Myogenic genes analysis in dystrophic models as compared to wild-type mice: (**a**) mRNA relative expression of Myf5, MyoD, Myog; Significant differences were observed for the Myog (Kruskal-Wallis test, Chi-square = 17.93, df = 3, p < 0.001) *Shows outliers animals. (**b**) MYOG western blot protein analysis (full blots figures without contrast adjustments are represented in Supplementary Material). N = 6 animals per strain, (**c**) Densitometric analysis of MYOG protein results. Significant differences were observed for the Myog gene ^#^p < 0.05 and ^##^p < 0.01 (Kruskal-Wallis test, Chi-square = 15.20, df = 3, p = 0.002), both at mRNA and protein levels.

We also investigated the relative expression of *Myf5*, *Myod*, *and Myog*, which are important genes of the myogenic program. No significant differences were found among the three dystrophic models and the wild-type regarding the expression of *MyoD* (Kruskal-Wallis Test *- Chi-square* = 3.22, *df* = 3, *p* = 0.359), nor *Myf5* (*Chi-square* = 3.21, *df* = 3, *p* = 0.361). In spite of that, we observed a significant elevation (*Chi-square* = 17.93, *df* = 3, *p* < 0.001) in *Myog* expression among the four mice models. Using the post hoc analysis with Bonferroni method of correction, a significant difference on *Myog* expression was found between wild-type and *Large*^*myd*^ (*p* < 0.001), wild-type and *DMD*^*mdx*^*/Large*^*myd*^ (*p* = 0.0023), and wild-type and *DMD*^*mdx*^ (*p* = 0.0067). No significant differences were found between the dystrophic strains (Fig. 4a). High *Myog* expression was confirmed by analysis of protein levels (Fig. 4b,c).

As data presented great variance in the expression of the myogenic factors, the non-parametric Levene’s test was done to verify the equality of variances in samples. We observed that they were not significantly different (*Myod*, *p* = 0.997; *Myf5*, *p* = 0.973; *Myog*, *p* = 0.190; *Pax7*, *p* = 0.890).

### Dystrophic muscle retains the ability to form new fibers

**Figure 5:**
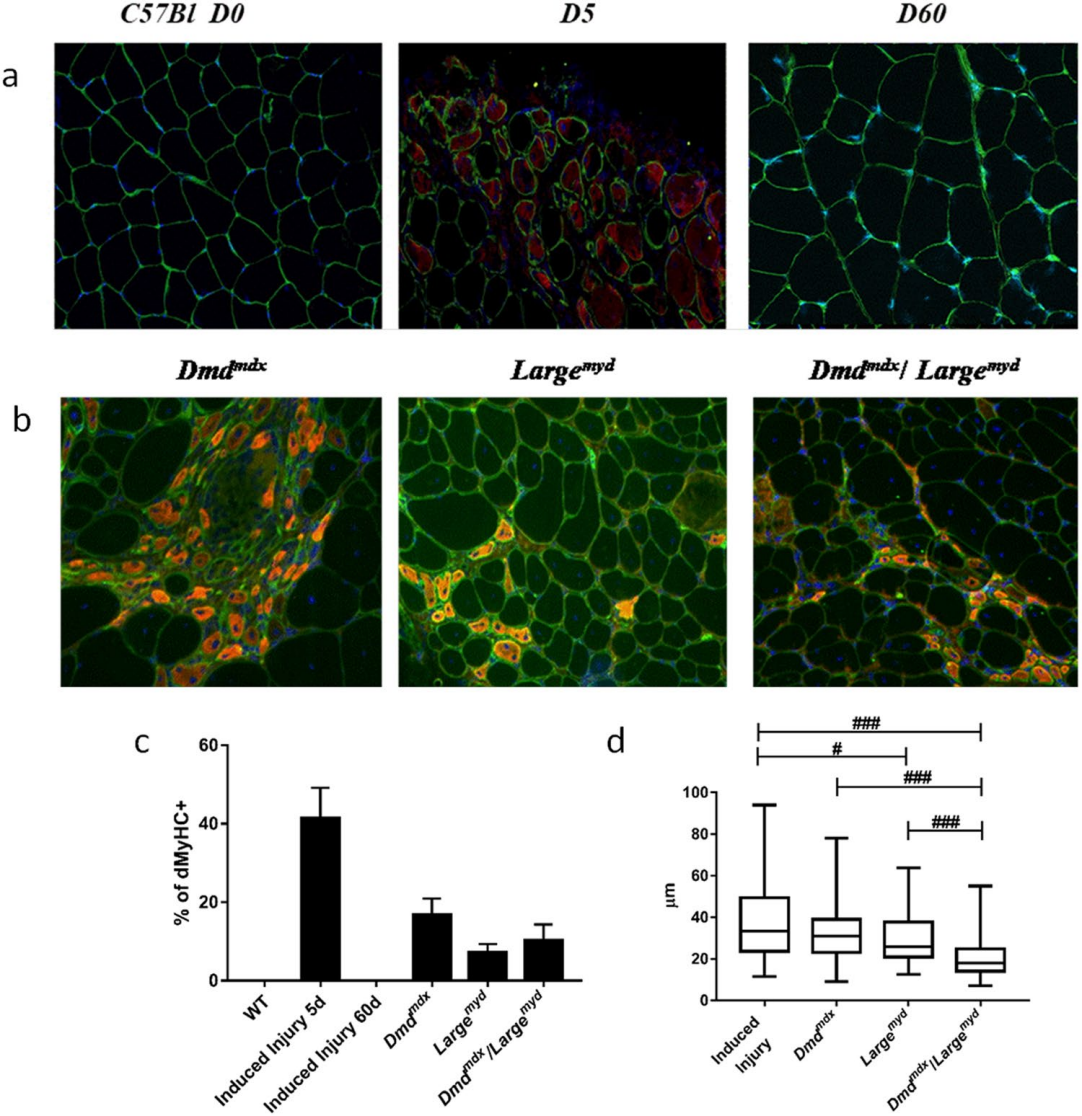
Representative images of dMyHC+ positive new regenerated fibers, normal and dystrophic muscles (full immunofluorescence figures are represented at Supplementary Material). dMyHC+ fibers are labeled in red/orange. Magnification 200X. (**a**) Immunofluorescence staining for dMyHC+ in normal WT muscle at time zero, 5 days after induced injury and after 60 days of induced injury, N = 6 animal per time. (**b**) dMyHC+ fibers in the 3 dystrophic models. N = 6 animals per strain (**c**) percentage of dMyHC+ fibers related to total fiber number (n = ~1500 fibers/animal). (**d**) dMyHC+ fiber diameter. Significant differences: ^#^p < 0.05 and ^###^p < 0.001 (Kruskal-Wallis test, Chi-square = 53.649, df = 3, p < 0.001).

The number and diameter of new muscle fibers were evaluated by detection of developmental myosin heavy chain isoform (dMyHC) (Fig. 5a,b). Immunofluorescence analysis showed no dMyHC+ fibers in wild-type muscles, while *Dmd*^*mdx*^ strain presented the greatest number of dMyHC+ fibers (17%). *Large*^*myd*^ and *Dmd*^*mdx*^*/Large*^*myd*^ showed respectively 7% and 10% of new fibers (Kruskal-Wallis Test, *Chi-square* = 13.87, *df* = 3, *p* = 0.003, Fig. 5c), which is compatible with an intense regeneration in all dystrophic strains. Interestingly, compared to normal mature fibers size (52 µm+/−10 µm), the diameter of these new fibers was smaller, with a mean of 33 µm ± 13.5 µm in *Dmd*^*mdx*^, 30 µm ± 12.1 µm in *Large*^*myd*^ and 20 µm ± 9.0 µm in *Dmd*^*mdx*^*/Large*^*myd*^ (Fig. 5d), with differences among the dystrophic strains (Kruskal-Wallis test, *Chi-square* = 53.649, *df* = 3, *p* < 0.001). In addition, the size of these new dMyHC+ fibers was more homogeneous, as shown by the coefficient of variation within normal values (lower than 250) in *Dmd*^*mdx*^ (150), *Large*^*myd*^ (245) and *Dmd*^*mdx*^*/Large*^*myd*^ (155). These results suggest that in the dystrophic models, these new fibers remain small and do not complete the regenerative process.

To confirm our results, the muscles from the different dystrophic strains were compared to regenerating normal muscle subjected to acute lesion by electroporation, at days zero, five, and 60 days post-injury (Fig. 5a). In wild-type animals, regenerating dMyHC+ fibers appeared five days post-injury and presented variable diameter. The coefficient of variation was very high (484), demonstrating the diversity in fiber diameter. After 60 days, no regenerating fiber was identified, and fiber diameter was more homogeneous^18^.

### Muscle degeneration is persistent in spite of regeneration

**Figure 6:**
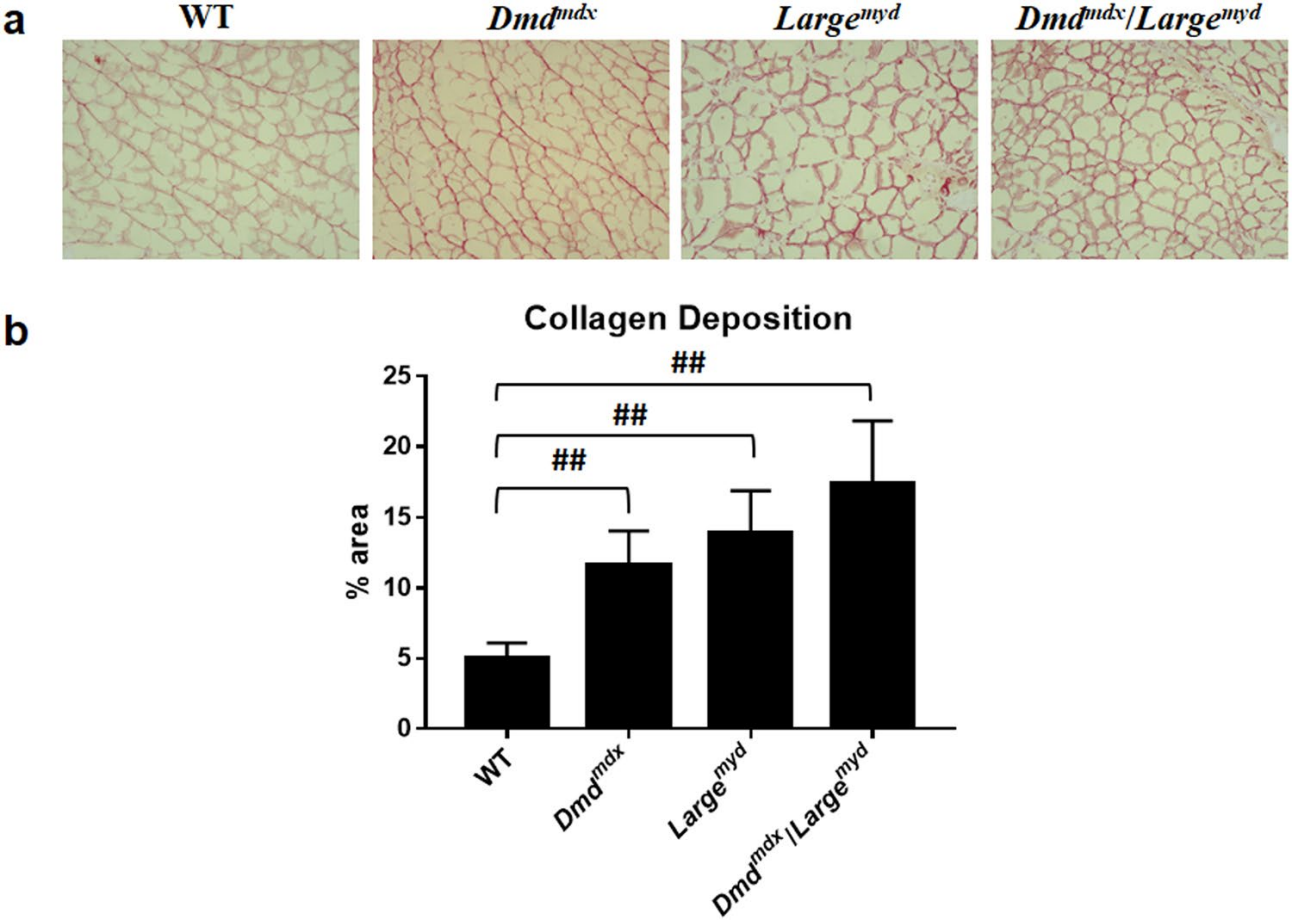
Connective tissue replacement in muscle degeneration (full Sirius red images are represented at Supplementary Material): (**a**) Representative images of collagen deposition by Sirius red staining of gastrocnemius muscles of WT, Dmd^mdx^, Large^myd^ and Dmd^mdx^/Large^myd^ mice, N = 6 animals per strain, magnification of 100X. (**b**) Quantification of percentage area of collagen deposition. Significant differences relative to WT muscle: ^##^p < 0.01 (Kruskal-Wallis test, Chi-square = 30.31, df = 2, p < 0.001).

The regeneration outcome was evaluated through the quantification of connective tissue substitution, using Sirius red staining (Fig. 6a). We observed more connective tissue infiltration in dystrophic mice than in wild-type mice (Kruskal-Wallis Test, *Chi-square* = 81.21, *df* = 3, *p* < 0.001). The differences among the three dystrophic strains were also statistically significant (Kruskal-Wallis Test – *Chi-square* = 30.31, *df* = 2, *p* < 0.001), with a higher proportion in the more severely affected muscles: *Dmd*^*mdx*^*/Large*^*myd*^ > *Large*^*myd*^ > *Dmd*^*md*.^ (Fig. 6b).

**Figure 7:**
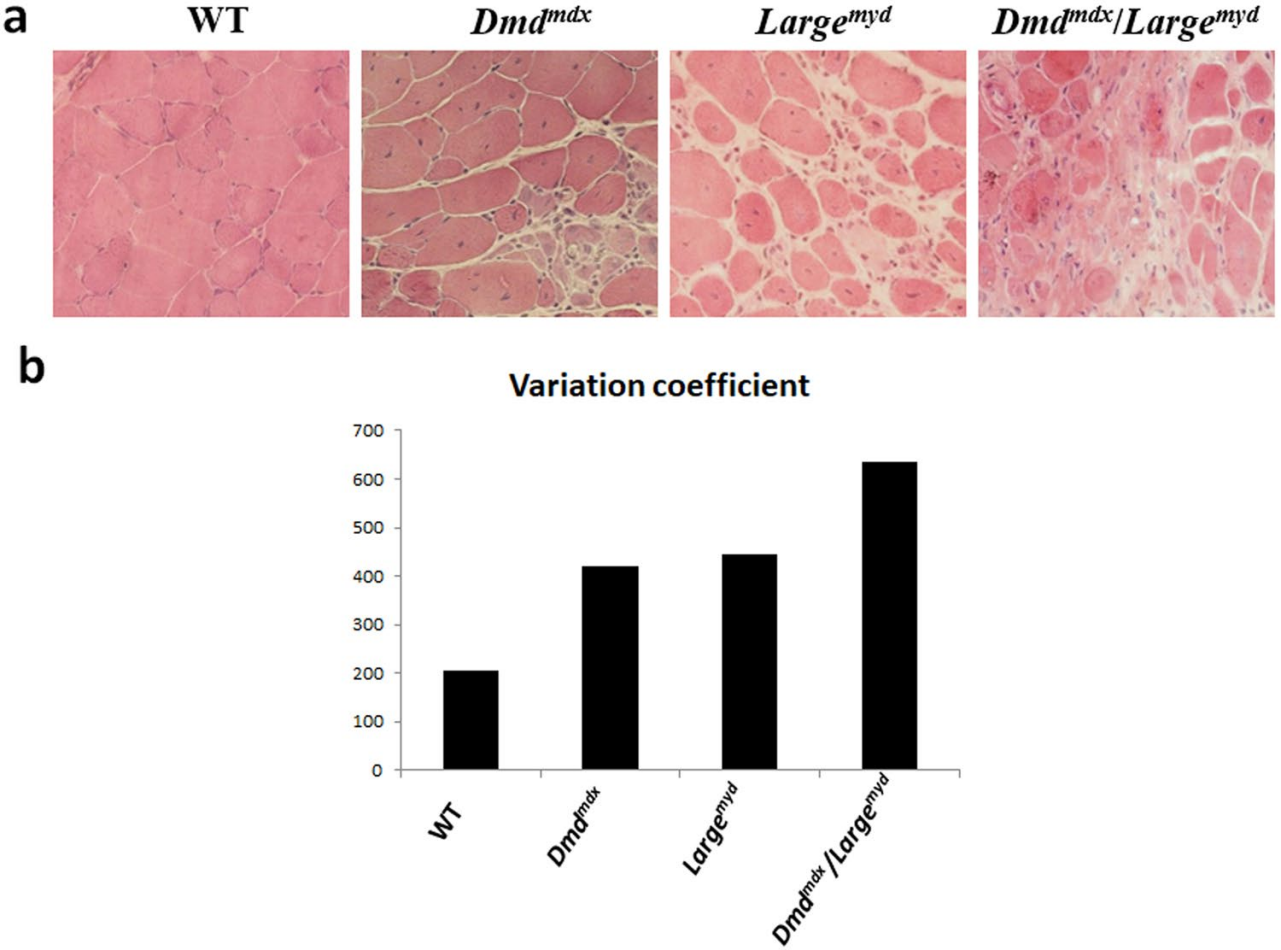
Histopathological alterations in the three dystrophic strains and wild-type (full H&E figures, without contrast adjustments, are represented at Supplementary Material). (**a**) HE staining, magnification 200X. N = 6 animals per strain. (**b**) Variation coefficient of muscle fiber size (>250 is considered pathogenic)^37^.

Histological analysis of muscles by H&E staining also showed a gradual increase in muscle degeneration and variation in fiber size (Fig. 7a). An increase in the variation coefficient of fiber size in dystrophic strains was observed (normal: up to 250), in agreement with the degree of muscle affection: 635 in *Dmd*^*mdx*^*/Large*^*myd*^ > 444 in *Large*^*myd*^ > 420 in *Dmd*^*mdx*^ > 205 in wild-type (Fig. 7b).

## Discussion

The reservoir of muscle precursor cells, the SCs, is responsible for after-birth growth and muscle regeneration as a response to injuries, either by exercise or disease. Since the first description in 1961 by Mauro^4^, SCs have been the target of a huge number of studies, aiming to understand their behavior and action. SCs are able to self-renew and replenish the stem cell pool, and this role is primordial for muscle regeneration. The regenerative response after muscle injury is marked by the exit of SCs from quiescence and their proliferation and fusion, leading to myofiber repair or new myofiber formation^19^. At the same time, there is the activation of an inflammatory response, with the infiltration of immune cells, mainly macrophages^19^. The dystrophic muscle is persistently injured due to a specific primary mutation in genes encoding for important muscle proteins, and the regenerative process is incessantly activated, recruiting SCs at higher rates than in normal tissue. Nevertheless, progressive replacement of muscle by fibrofatty tissue in dystrophic muscle has been considered as a factor that impairs the complete regeneration process^20–22^.

Many studies have suggested that the regenerative capacity of SCs would be finite, and exhaustion of the pool of these cells has been considered a significant factor that contributes to progressive muscle deterioration in muscular dystrophies, as studied in DMD^5,23^ and in mdx/utrophin^−/−^ mice^24^. SCs repair potential, therefore, would not be sufficient to compensate for the persistent degeneration process^25^. A recent study showed that deficiency of DMD protein could lead SCs to divide abnormally, resulting in the depletion of myogenic progenitors. In the long term, this condition can impair muscle regeneration^26^. In contrast, several studies have shown an increased number of SCs in human and mice dystrophic muscles^20–22,27^. The dystrophic niche seems also to be crucial, since removing SCs from a dystrophic milieu revealed that their regenerative capacity remains both intact and similar to SCs derived from healthy muscle^28,29^. Moreover, as observed in dystroglycanopathies, muscle fibrosis increases laminin disorganization, and this alteration in extracellular matrix composition is suggested to impair SCs function and activity^30^.

To elucidate the players and mechanisms involved in SCs function is of extreme importance, especially for therapeutic strategies. Here, we studied SCs protein and gene expression in different forms of murine muscular dystrophies, with a specific pattern of muscle histopathology, to correlate the impact of muscle degeneration in the population of SCs in these different microenvironments. The expression of the transcription factor PAX7 by SCs has been correlated with the maintenance of these cells in the undifferentiated state, being an important factor for SCs self-renewal that can be expressed both by quiescent and by early activated SCs^31^. We studied *Pax7* mRNA expression in the muscle of dystrophic mouse models, as compared to wild-type, to determine if there was a reduction in SCs population with the dystrophic process. We also identified and counted PAX7-positive SCs in muscle sections and verified the number of activated cells among them in each mouse model. Our analyses, through mRNA expression, protein quantification, and immunohistochemical evaluation showed similar results in all strains, suggesting the maintenance of a normal pool of SCs despite the active but diverse process of regeneration and degeneration that occurs in the *Dmd*^*mdx*^, *Large*^*myd*^ and *Dmd*^*mdx*^*/Large*^*myd*^. These data are in accordance with the hypothesis of the maintenance/increased number of SCs with the dystrophic process^16,18,24^.

The importance of the niche for the regenerative capacity of SCs is well documented in the *Large*^*myd*^ mouse, in which SCs were present in a significantly higher number, but with reduced proliferation capacity when in native muscle fibers. However, when removed from their niche, their proliferative capacity in culture was restored^30^. This could also be observed in our models, with activated SCs present in the *Dmd*^*mdx*^, *Large*^*myd*^ and *Dmd*^*mdx*^*/Large*^*myd*^ in a similar proportion, and higher than in wild-type. This self-renewal capacity of SCs, even in the dystrophic muscle, is extremely important for the replenishment of the stem cell pool, mainly in an attempt to regenerate^32^.

The molecular players of regeneration are the muscle regulatory factors, including MYF5 and MYOD, responsible for muscle-cell type determination and SCs activation, and MYOG, responsible for muscle differentiation^33,34^. Thus, we measured the relative expression of these genes in our three different dystrophic models. At the mRNA level, we observed similar relative expression of *Myf5* and *Myod* in all strains and higher expression of *Myog* in the three dystrophic strains as compared to wild-type, suggesting activation of a later stage of muscle regeneration in all of them, independently of the muscle degeneration degree.

MYF5 is expressed in both activated and quiescent SCs, but quiescent cells can maintain their quiescence state using a post-transcriptional regulation by miR-31, which sequester *Myf5* mRNA in messenger ribonucleoprotein (mRNP) granules. Upon activation, miR-31 levels decrease and *Myf5* mRNA is released to translation^35^. Therefore, this could explain the similar relative quantification of *Myf5* mRNA in all strains.

MYOD can be expressed both in first steps of SCs differentiation and by myocytes in the final steps of differentiation^1,33,36^. Upregulation of MYOD expression leads SCs to exit the cell cycle and to differentiate and fuse, forming myotubes^33^. In spite of the normal expression of MYOD in the muscle of these three dystrophic models, they showed regenerating fibers as observed by dMyHC staining (Fig. 5). This data confirms the maintenance of SCs capacity to follow a myogenic program in the dystrophic muscle and to be able to form new fibers, as already observed in *Dmd*^*mdx* 28^.

In the final step of myogenesis, myogenin expression has a key role in the differentiation of SCs into myotubes. High expression of myogenin initiates myoblast maturation to myocytes, myocytes fusion and myotube formation, a process extremely active in dystrophic muscle^2,33^. We studied MYOG expression in the dystrophic strains to investigate whether myotube formation is impaired in these models. Gene expression analysis showed a significant increase of *Myog* expression as well as protein expression in the three dystrophic strains, suggesting the occurrence of myoblast maturation to myocytes, fusion induction, and myotube formation in all the dystrophic muscles^37^.

Newly regenerated muscle fibers have particular characteristics such as central myonuclei, a basophilic pattern on H&E staining, and expression of developmental myosin heavy chain (dMyHC) isoform^38^. In our analysis of regenerating fibers expressing dMyHC, we observed a significant number of new fibers in all the dystrophic strains, including the most severely affected double mutant *Dmd*^*mdx*^*/Large*^*myd*^. The *Dmd*^*mdx*^ strain showed the highest number of regenerating fibers, which is compatible with its pronounced regenerative capacity and milder dystrophic phenotype. *Large*^*myd*^ and *Dmd*^*mdx*^*/Large*^*myd*^ strains also presented a significant amount of regenerating fibers in their muscle, in spite of the more severe dystrophic phenotype, suggesting that the formation of new fibers is possible even when the muscle is already significantly degenerated.

Contrarily, all dystrophic strains showed, on average, thinner regenerating fibers, especially the double mutant *Dmd*^*mdx*^*/Large*^*myd*^, as compared to the other strains (Fig. 5d). These results suggest that in a harsher dystrophic microenvironment, the complete regeneration can be compromised. In addition, the fiber size variation coefficient^38^ showed small variation, which suggests the maintenance of these regenerating fibers in an immature state with small diameter, not progressing to a normal mature fiber size. The possibility of fiber branching or splitting fibers cannot be totally ruled out, since it is an alteration commonly found in dystrophic muscles, happening both in degeneration and regeneration conditions. Branched fibers were well described mainly in old fast-twitch EDL muscle approaching the end stage of the dystrophic muscle disease due to dystrophin deficiency^39^. During pathological conditions, splitting or branching of large fibers can lead to the formation of many small fibers, increasing fiber diameter variability. The regenerative process can also originate small fibers, but here, we confirmed their immaturity through dMyHC expression.

Next, we compared these data with the regeneration process of normal muscle under induced acute lesion by electroporation. At five days post-injury, an intense regeneration was observed with the presence of a high number of dMyHC+ muscle fibers. These new normal fibers presented more variable diameter, leading to a higher variation coefficient. This occurs because these fibers are probably ongoing a normal process of regeneration, reaching an increased size 60 days post-lesion^18^.

Taken together, these results suggest that the three dystrophic models retain their regenerative capacity, including the maintenance of SCs pool, the proliferation capacity, differentiation marker expression and finally, the formation of new fibers. However, this regenerative process is incomplete, with a possible defect in the maturation characteristics that could be determinant to the dysfunction of these fibers.

Remarkably, very interesting data, which are in accordance with our proposal, has been shown by Pipalia *et al*.^40^ in a model of wound repair in larval zebrafish. Two different populations of Pax7+ stem cells with different roles were observed: one, expressing Pax7a, which only initiates fiber formation, and the second, formed by Pax7b cells, which contribute to fiber growth. These two myoblast populations suggest a novel cellular complexity in muscle repair, with the possibility of the presence of distinct populations of myogenic cells contributing differentially to the repair also in other vertebrates. The dystrophic process could alter more specifically the second cell type, associated with fiber growth post-regeneration, and explain the presence of smaller regenerating fibers in the mouse models for muscular dystrophies.

As to the degenerative process, we studied histopathological alterations through both connective tissue replacement and variation in fiber size, as a correlation with their regenerative potential. Variability in the coefficient of muscle fiber size >250 is considered pathogenic^38^, and this was observed in all dystrophic strains. We also observed more connective tissue infiltration in dystrophic mice, with a crescent increase in muscles with more weakness, such as *Large*^*myd*^ and *Dmd*^*mdx*^*/Large*^*myd*^. Thus, we conclude that the active regeneration in the dystrophic muscle is not indicative of effective improvement of muscle health, but an insufficient attempt to restore muscle fibers.

In conclusion, our findings suggest that dystrophic muscles, independently of the degree of degeneration caused by the primary gene defect, retain the pool of SCs with proliferating capacity and are ready to respond to regenerating stimuli. The potential to repair is preserved by the upregulation of myogenic factors like MYOG and by the formation of new fibers expressing dMyHC. Nonetheless, the maturation of these new fibers is incomplete, since these fibers remain small and do not prevent muscle degeneration, as observed by the intense substitution by connective tissue. In this sense, efforts to improve late muscle regeneration should better contribute to therapeutic approaches.

## Methods

### Animals

Histological, immunofluorescence, gene expression and protein analyses, were examined in the gastrocnemius muscle of adult male mice with 12–14 weeks of age. This muscle has about 51.5% of fiber type IID, 22.7% of fiber type IIA, 18.2% fiber type IIB and 7.6% of fiber type I^41^. The studied strains were: *DMD*^*mdx−*^ (n = 6), *Large*^*myd*^ (n = 6), *C57Bl/6* (n = 6) and double mutant *DMD*^*mdx*^/*Large*^*myd*^ (n = 6, three males and three females).

During the experiments, animals were bred and housed in controlled temperature and light in the animal house of the Human Genome and Stem Cell Research Center (HUG-CELL).

All experimental protocols and methodologies were performed in accordance with and were approved by CEUA - COMISSÃO DE ÉTICA NO USO DE ANIMAIS (Ethical committee of the use of animal in experiments - http://www.ib.usp.br/comissoesib/ceua/), of the Institute of Biosciences, University of Sao Paulo (protocol number 246/2016), which is the licensing committee of our Institution. In addition, the used methods were carried out in accordance with the relevant guidelines and regulations from the Brazilian National Council for Control of Animal Experimentation (Conselho Nacional de Controle de Experimentação Animal – CONCEA).

### Tissue collection

After euthanasia, the gastrocnemii muscles were collected and frozen in liquid nitrogen to study gene expression, histopathology, immunofluorescence,and protein quantification.

### RNA extraction, cDNA synthesis, and qRT-PCR

RNA extraction from frozen muscles was performed using the RNeasy Microarray Tissue Mini Kit (Qiagen). RNA was solubilized in RNase-free water (Ambion™) and quantified in a Nanodrop spectrophotometer (Thermo Fisher Scientific). The cDNA synthesis was performed using 1 µg of total RNA according to the M-MLV protocol (Invitrogen). Samples were amplified in triplicates using the MasterMix containing Sybr Green (Applied Biosystems) and primers (Table 1) on a thermocycler 7500 Fast (Applied Biosystems). The fold-change was obtained by the 2^−ΔΔCT^ method, with *Tbp* gene as normalizer.

**Table 1:** Primers sequences.

Gene	Forward sequence	Reverse sequence
*Pax7*	5′ GAGTTCGATTAGCCGAGTGC 3′	5′ GAGTTCGATTAGCCGAGTGC 3′
*Myf5*	5′ GAAGGTCAACCAAGCTTTCG 3′	5′ GCTCTCAATGTAGCGGATGG 3′
*MyoD*	5′ TACAGTGGCGACTCAGATGC 3′	5′ TAGGCGGTGTCGTAGCC 3′
*Myog*	5′ CTGCACTCCCTTACGTCCAT 3′	5′ CCCAGCCTGACAGACAATCT 3′
*Tbp*	5′ TGCACAGGAGCCAAGAGTGAA 3′	5′ CACATCACAGCTCCCCACCA 3′

### Protein analysis

Frozen muscle samples were homogenized in RIPA buffer (Sigma-Aldrich) supplemented with 1% of protease inhibitors (Sigma-Aldrich). Samples were kept on ice for 30 minutes and afterward centrifuged at 16.000 g for 20 min at 4 °C, followed by protein quantification using the Pierce BCA Assay kit (Thermo Scientific). Homogenized samples with 30 µg of protein were separated by SDS-PAGE in 4–15% Mini-PROTEAN® TGX™ Gels (BioRad) and transferred to nitrocellulose membranes using iBlot® Gel Transfer Device (Invitrogen) for 7 minutes. The obtained membranes were probed using the antibodies: PAX7 (1:1000 Novus Biologicals; NBP2-34706) and Myogenin (Abcam ab15232)^42^. The membranes were then incubated with the appropriated secondary horseradish peroxidase conjugated anti-rabbit or anti-mouse antibodies (Thermo Fisher Scientific) and revealed using the Novex™ ECL Chemiluminescent Substrate Reagent kit (Invitrogen). Images were obtained using photo-documenter (ImageQuant TL, GE Healthcare Life Sciences), all images were collected after 450 seconds of standard exposure. For the reaction with the endogenous control, membranes were stripped with a stripping buffer (Thermo Fisher Scientific) and reprobed with GAPDH antibody (1:1000 Cell Signaling 2118)^43^. Bands were quantified by densitometry using the software ImageQuant TL (GE Healthcare Life Sciences). The protein of interest and the endogenous protein were probed and analyzed in the same membrane, both for experimental and control samples.

### Histology and immunofluorescence analyses

#### Hematoxylin-eosin (H&E)

Transverse cross sections of the right gastrocnemius were stained with H&E and histopathological evaluation was performed by measuring muscle fibers to obtain the fiber diameter utilizing ImageJ software (n = 100 fibers/experimental group). The mean fiber diameter of each strain was obtained, and the variability coefficient was calculated according to the formula: Standard Deviation x 1000 ÷ Diameter Mean. Results were represented as mean variation and variability coefficient. The stained sections were observed under a light microscope (Zeiss).

#### Sirius red staining

To identify regions with collagen deposition, indicative of connective tissue replacement and infiltration between muscle fibers, the slides were immersed in Bouin fixative for twenty minutes and washed in water. The sections were stained with Sirius red (0.2 g) dissolved in saturated picric acid (100 mL) for 60 minutes, dehydrated and mounted in Canada balsam. The differences were quantified by the positive area of staining, labeled in red, relative to the total area of the section, using ImageJ software (4–5 fields, n = 6 animals/experimental group). The stained sections were observed and photographed under the same exposure conditions (16 ms), using a light microscope (Zeiss) and the Axiovision software Release 4.8.2 SP3 (2013).

#### PAX7 and KI67 immunofluorescence

The presence of SCs was determined by double staining with antibodies against PAX7 and laminin to label the basal lamina. Activated SCs were determined by double staining with antibodies against PAX7 and KI67. Primary antibodies used: PAX7 (1:20; DSHB), laminin (1:100; Dako; Z0097) and KI67 (1:200; Abcam AB15580). Secondary antibodies were Cy3-labeled sheep anti-mouse IgG (1:100; Sigma; C2181) and FITC-labeled donkey anti-rabbit IgG (1:100; Amersham; N1034). The 6 µm muscle sections were fixed with 4% formaldehyde solution and submitted to antigen retrieval with hot citric acid buffer. Primary antibodies were incubated overnight at 4 °C. Secondary antibodies were incubated for one hour at room temperature. The slides were mounted with Vectashield with DAPI to counterstain nuclei. Immunofluorescence micrographs were obtained with a conventional fluorescence microscope (Zeiss). The proportion of PAX7 positive nuclei to the total number of fiber was quantified (~3000 fibers, n = 4 animals/experimental group). The proportion of double stained PAX7 and KI67 were analyzed in approximately 200 PAX7 positive nuclei. All images were collected using a Zeiss microscope with epi-fluorescence, using specific filters for DAPI, FITC and for Cy3. The images were photographed using the Axiovision software Release 4.8.2 SP3 (2013), under the same exposure conditions (120–130 ms), standardized for the Cy3 staining for identification of dMyHC expression. The same software created merged figures automatically.

#### Developmental myosin heavy chain (dMyHC)

The antibody Clone RNMy2/9D2 recognizes a myosin heavy chain (MHC) present during the embryonic and neonatal period in the development of skeletal muscle. The same MHC is expressed during regeneration of muscle fibres. The presence of dMyHC indicates myogenic activity by newly differentiated fibers labeled in red. The 6 µm muscle sections were double labeled with primary antibodies to dMyHC (1:30; Vector; VP- M664)^44^ and laminin (1:50; Dako; Z0097)^45^, washed, and then incubated for 1 h with secondary antibodies Cy3-labeled sheep anti-mouse IgG (1:100; Sigma; C2181) and FITC-labeled donkey anti-rabbit IgG (1:100; Amersham; N1034), respectively. After washing, the slides were mounted with Vectashield and DAPI, to counterstain nuclei. Immunofluorescence micrographs were obtained with a conventional fluorescence microscope (Zeiss) and the percentage of dMyHC+/total fibers was quantified (4 fields, n = 6 animals/experimental group). The fiber diameter of dMyHC+ fibers of each strain was measured manually, and the variability coefficient was calculated according to the formula: Standard Deviation x 1000 ÷ Diameter Mean^38^. All images were collected using a Zeiss microscope with epi-fluorescence, using specific filters for DAPI, FITC and for Cy3. The images were photographed using the Axiovision software Release 4.8.2 SP3 (2013), under the same exposure conditions (120–130 ms), standardized for the Cy3 staining for identification of dMyHC expression. Merged figures were created automatically by the same software.

### Statistical analysis

Differences among the groups were assessed using one-way ANOVA Test, and Kruskal-Wallis Test, for small and non-normally distributed set of data. Results are reported showing means and standard deviations in bar graphs and medians in boxplot graphs. The statistical analyses were calculated using GraphPad Prism version 7 for Windows (San Diego, CA: GraphPad Software, Inc), Minitab 17 Statistical Software (State College, PA: Minitab, Inc) and IBM SPSS Statistics for Windows version 24.0 (Armonk, NY: IBM Corp) software and a p-value equal or less than 0.05 was considered to be significant. For the expression analysis, additional five animals (males) from each strain were added (in a total of eleven animals), to confirm and strengthen the statistical analysis.

## Supplementary information

Supplemental information
