# Supplementary material for: Muscle satellite cells and impaired late stage regeneration in different murine models for muscular dystrophies
Source: Sci Rep. 2019 Aug 14;9:11842. doi: 10.1038/s41598-019-48156-7 (PMC6694188; doi:10.1038/s41598-019-48156-7)

**Supplementary information**

**Muscle satellite cells and impaired late stage regeneration in different murine models for muscular dystrophies**

Antonio F. Ribeiro Junior^1^, Lucas S. Souza^1^, Camila F. Almeida^1^, Renata Ishiba^1^, Stephanie A. Fernandes^1^, Danielle A. Guerrieri^1^, André L. F. Santos^1^, Paula C. G. Onofre-Oliveira and Mariz Vainzof^1^*.

### Affiliations

**^1^**Human Genome and Stem-cell Research Center, Biosciences Institute, University of São Paulo, São Paulo, 05508-090, Brazil.

*[mvainzof@usp.br](mailto:mvainzof@usp.br)

This supplementary information file contains 24 supplemental figures

**Supplemental Figure 1.** Immunofluorescence of WT mouse for PAX7 (red), green labeling for Laminin and blue labeling for nuclei. PAX7 positive cells stain in bright pink. Merged images. Magnification 200X.


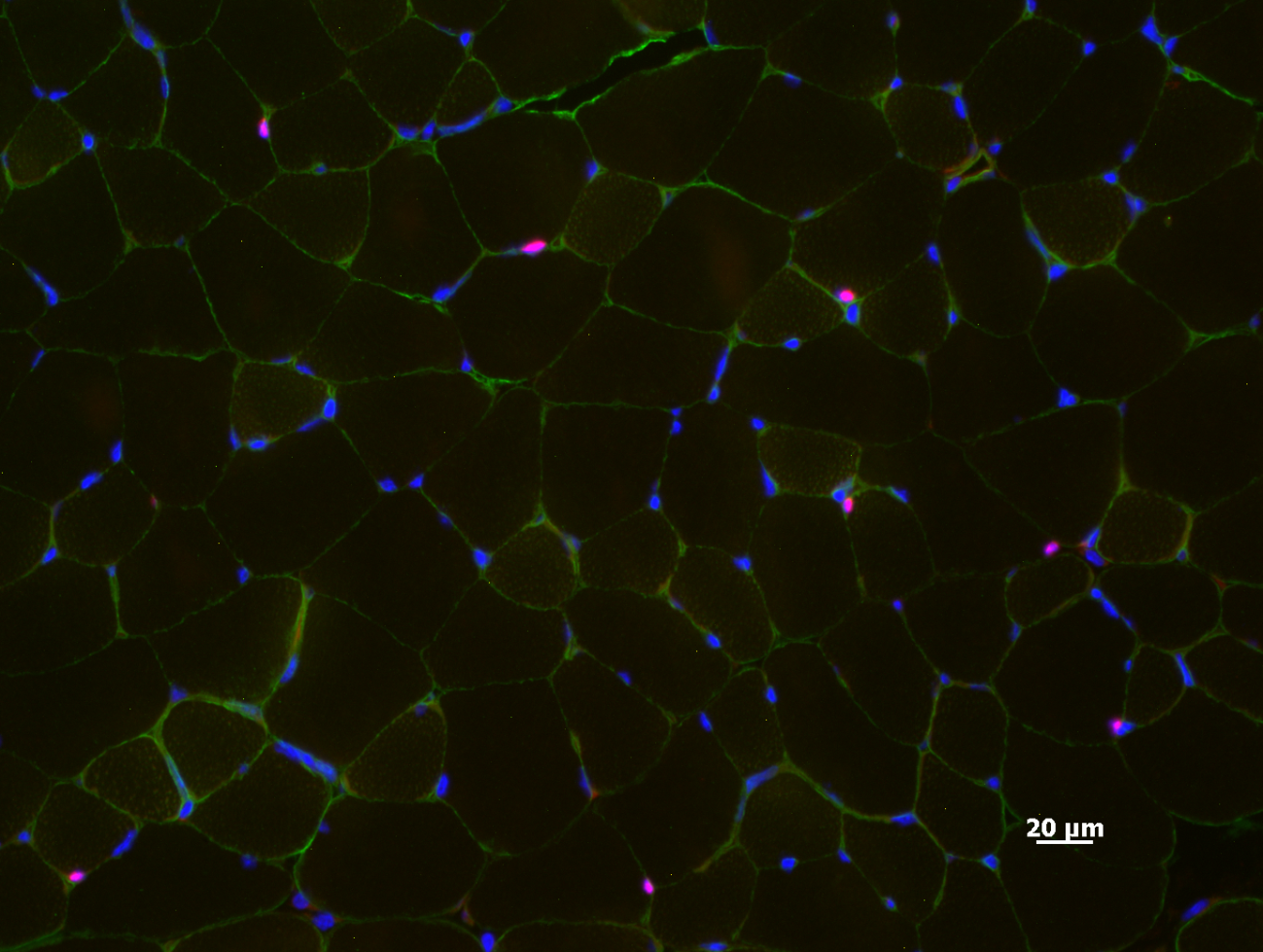


**Supplemental Figure 2.** Immunofluorescence of *Dmd^mdx^* mouse for PAX7 (red), green labeling for Laminin and blue labeling for nuclei. PAX7 positive cells stain in bright pink. Merged images. Magnification 200X.


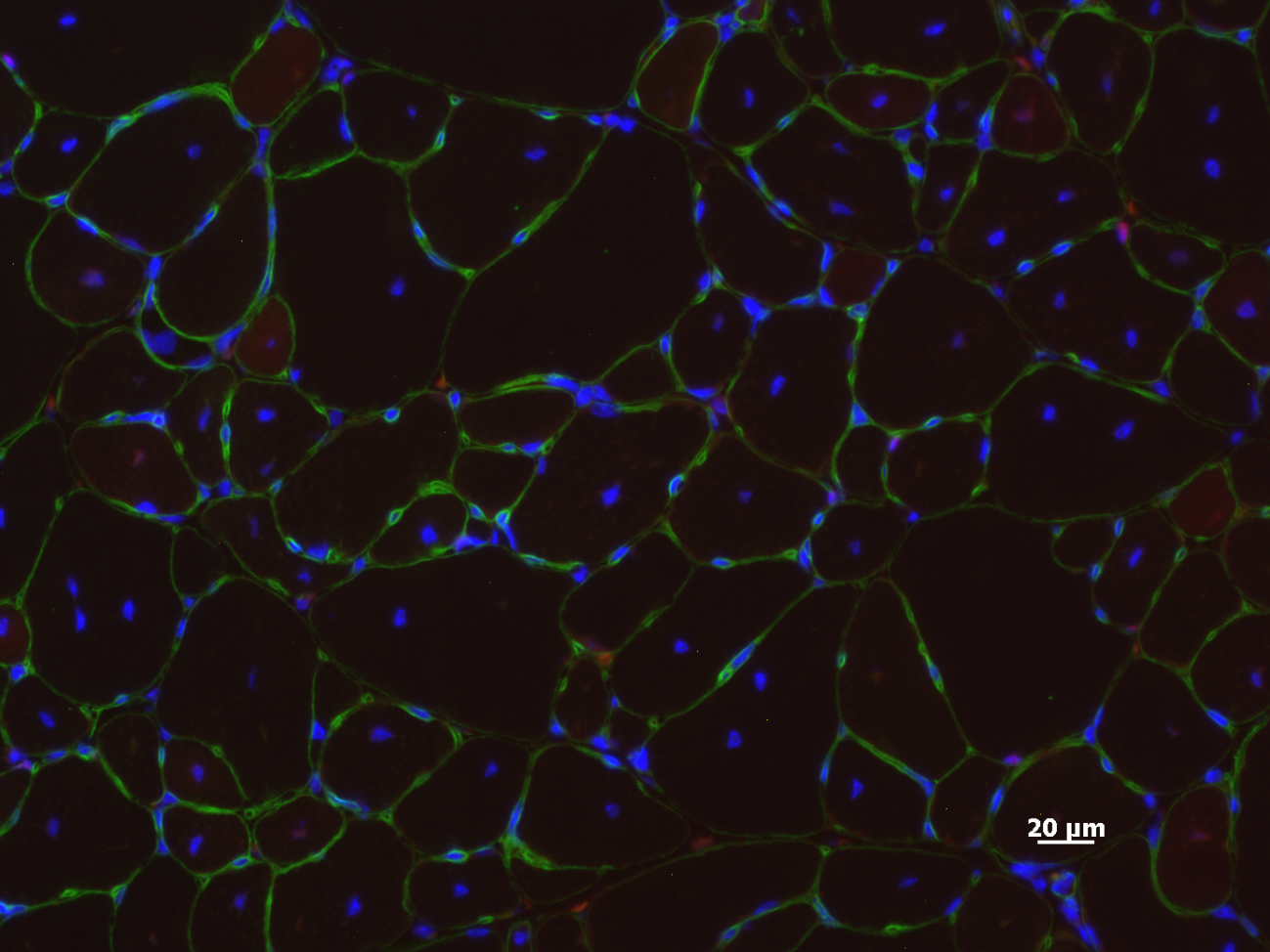


**Supplemental Figure 3.** Immunofluorescence of *Large^myd^* mouse for PAX7 (red), green labeling for Laminin and blue labeling for nuclei. PAX7 positive cells stain in bright pink. Merged images. Magnification 200X.


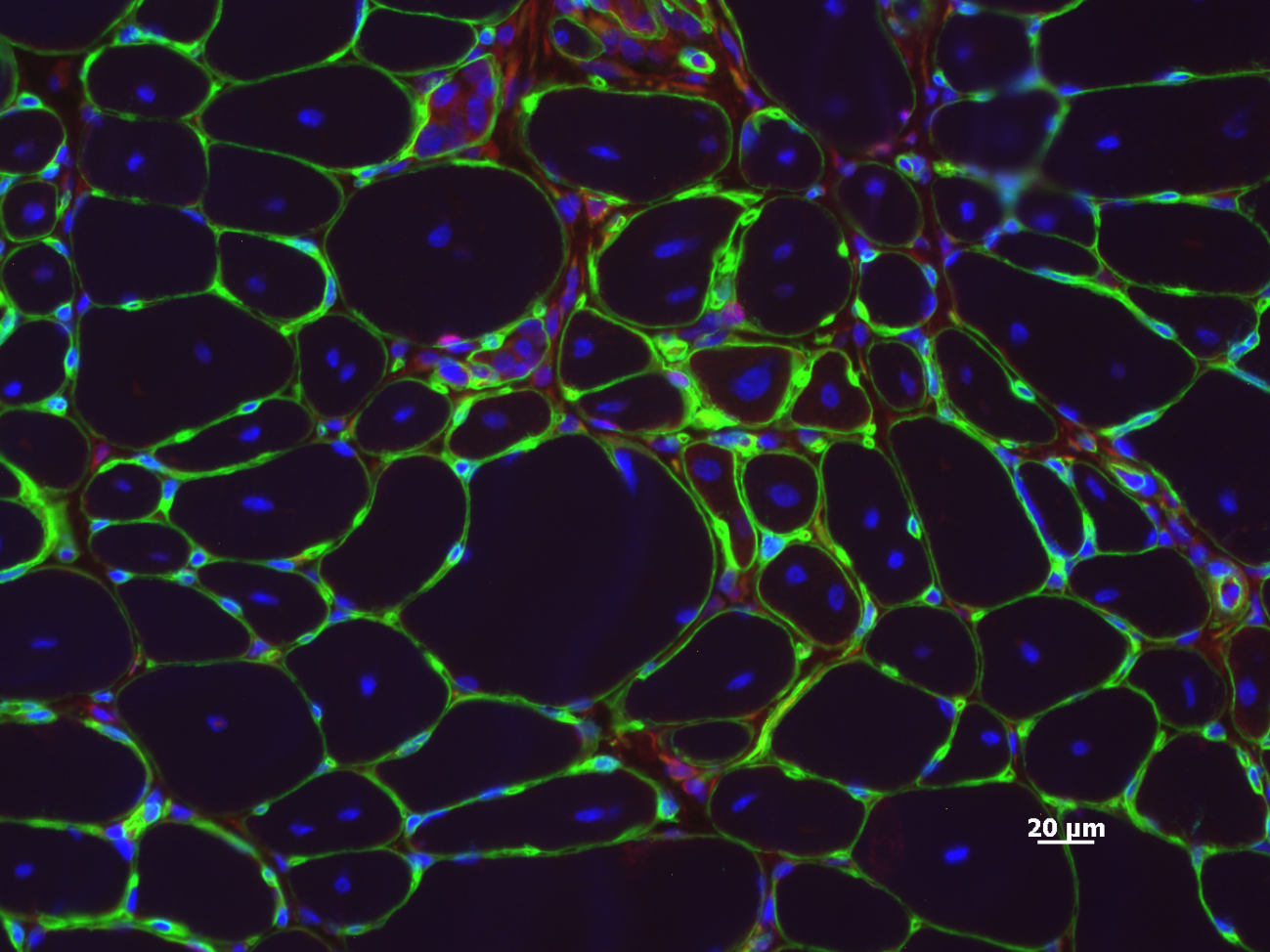


**Supplemental Figure 4.** Immunofluorescence of *Dmd^mdx^/Large^myd^* mouse for PAX7 (red), green labeling for Laminin and blue labeling for nuclei. PAX7 positive cells stain in bright pink. Merged images. Magnification 200X.


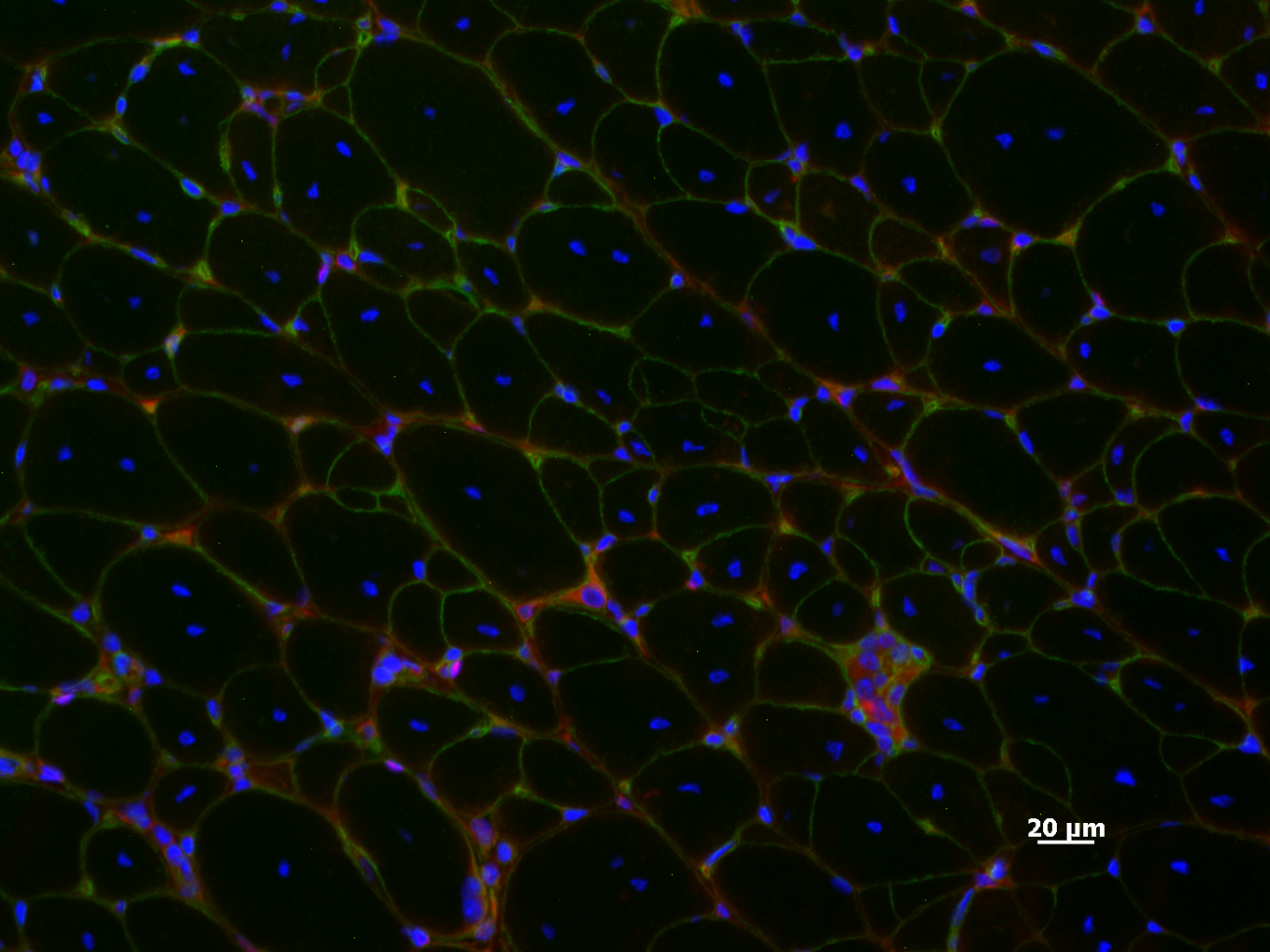


**Supplemental Figure 5.** Immunofluorescence of *Large^myd^* mouse for PAX7 (red), green labeling for Ki67 and blue labeling for nuclei. PAX7 and Ki67 positive cells stain in yellowish green. Merged images. Magnification 200X.


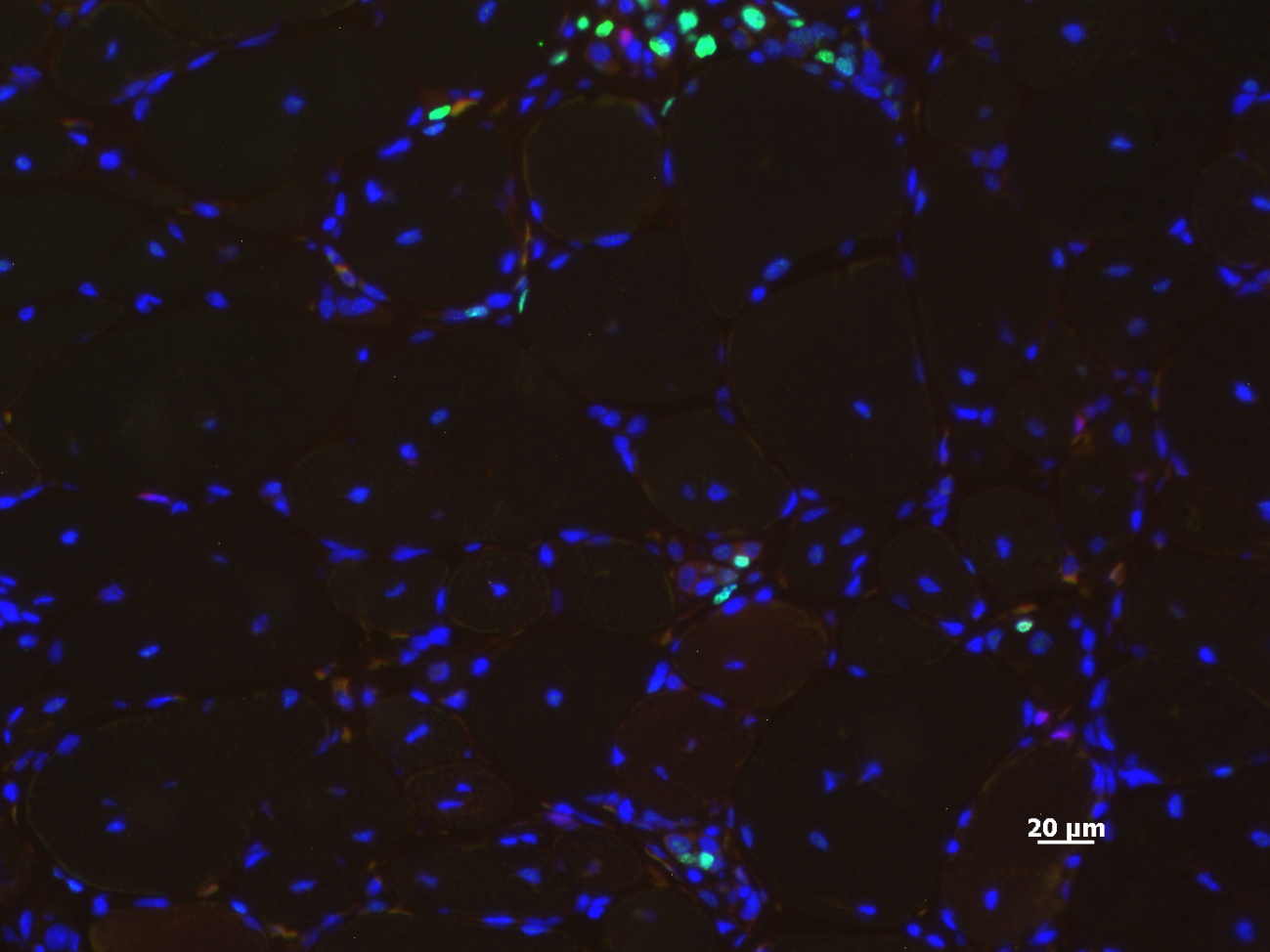


**Supplemental Figure 6.** Immunofluorescence of *Large^myd^* mouse for PAX7 (red), green labeling for Ki67 and blue labeling for nuclei. PAX7 and Ki67 positive cells stain in yellowish green. Blue channel image only. Magnification 200X.


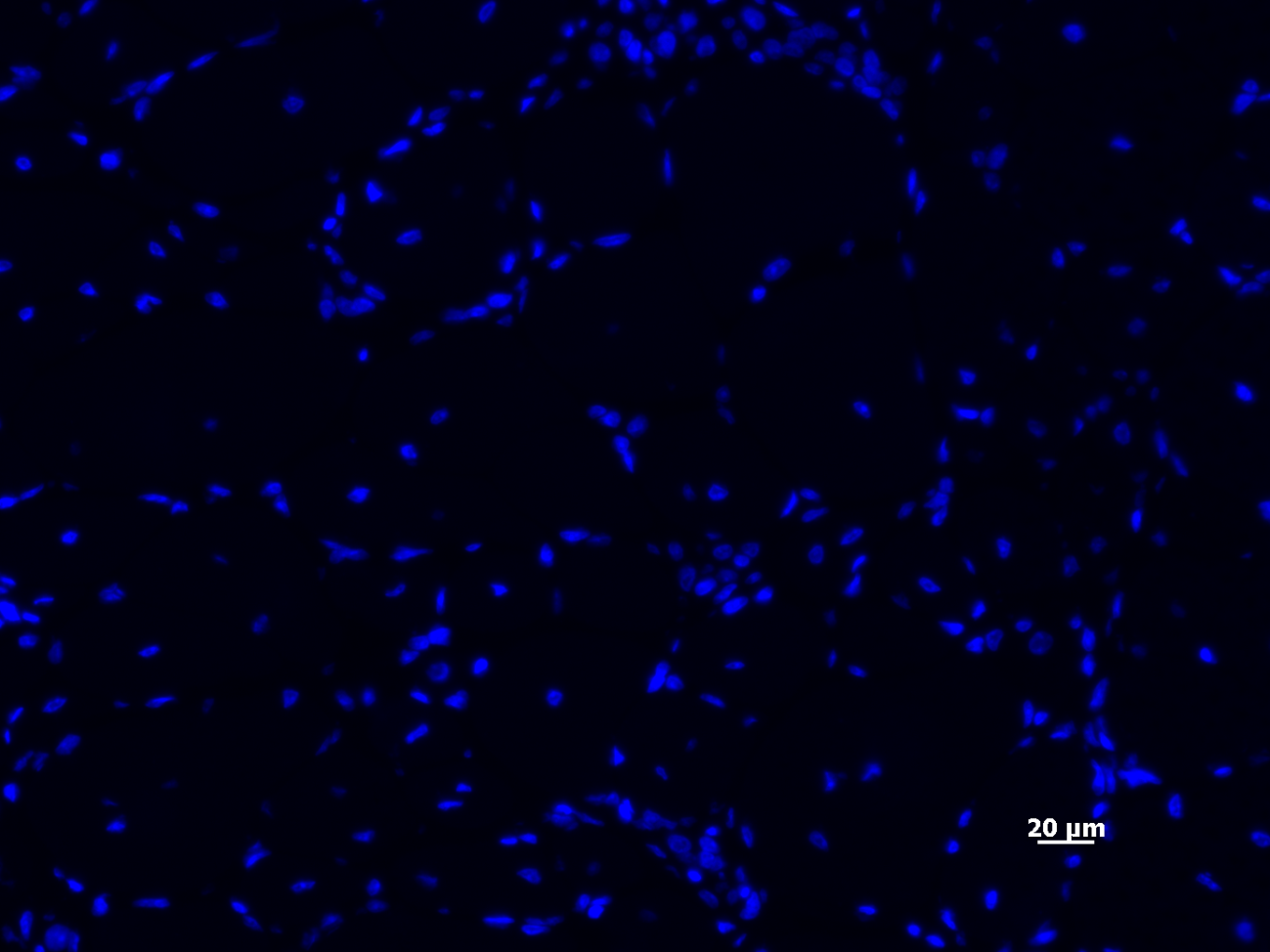


**Supplemental Figure 7.** Immunofluorescence of *Large^myd^* mouse for PAX7 (red), green labeling for Ki67 and blue labeling for nuclei. PAX7 and Ki67 positive cells stain in yellowish green. Red channel image only. Magnification 200X.


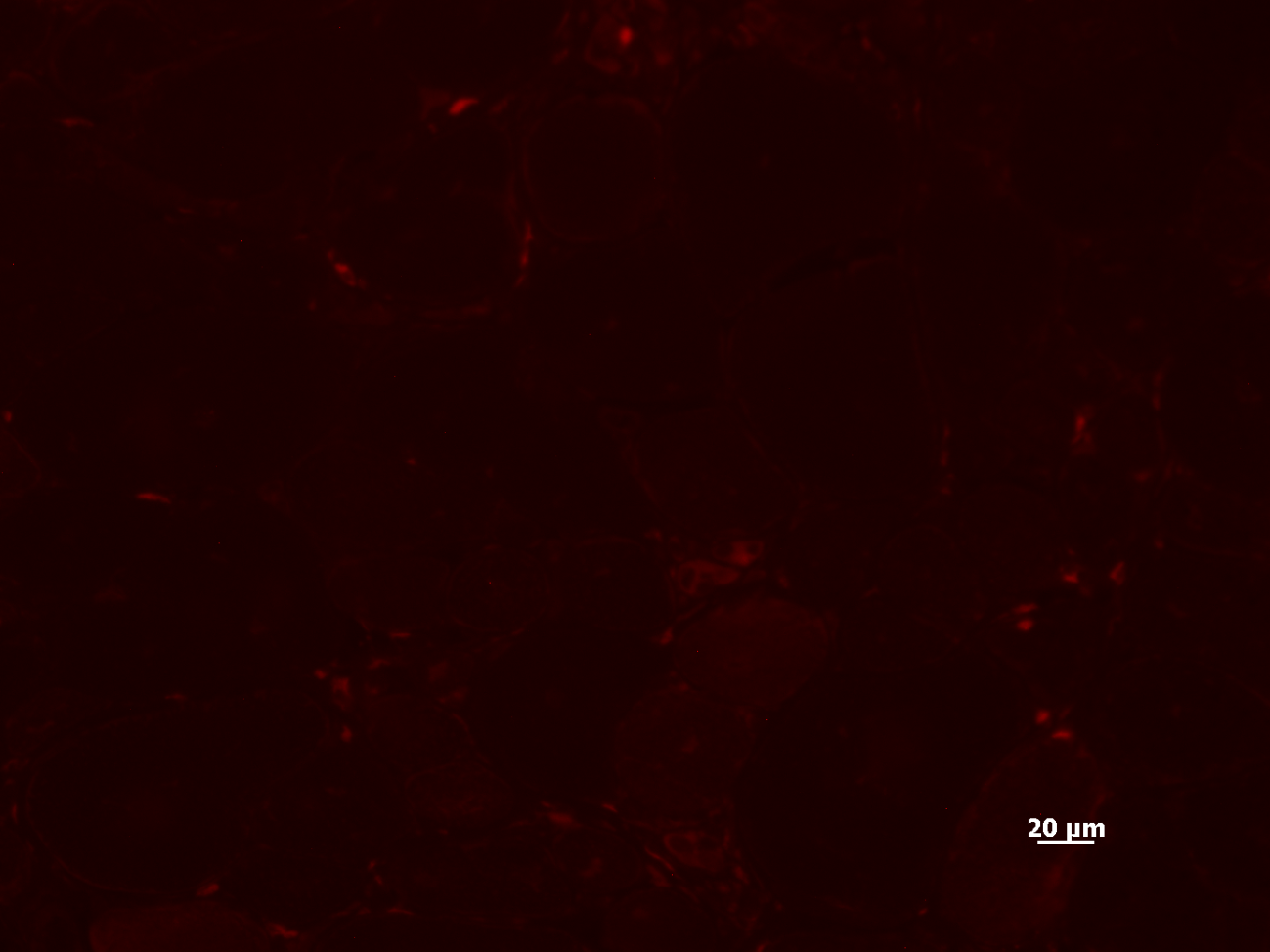


**Supplemental Figure 8.** Immunofluorescence of *Large^myd^* mouse for PAX7 (red), green labeling for Ki67 and blue labeling for nuclei. PAX7 and Ki67 positive cells stain in yellowish green. Green channel image only. Magnification 200X.


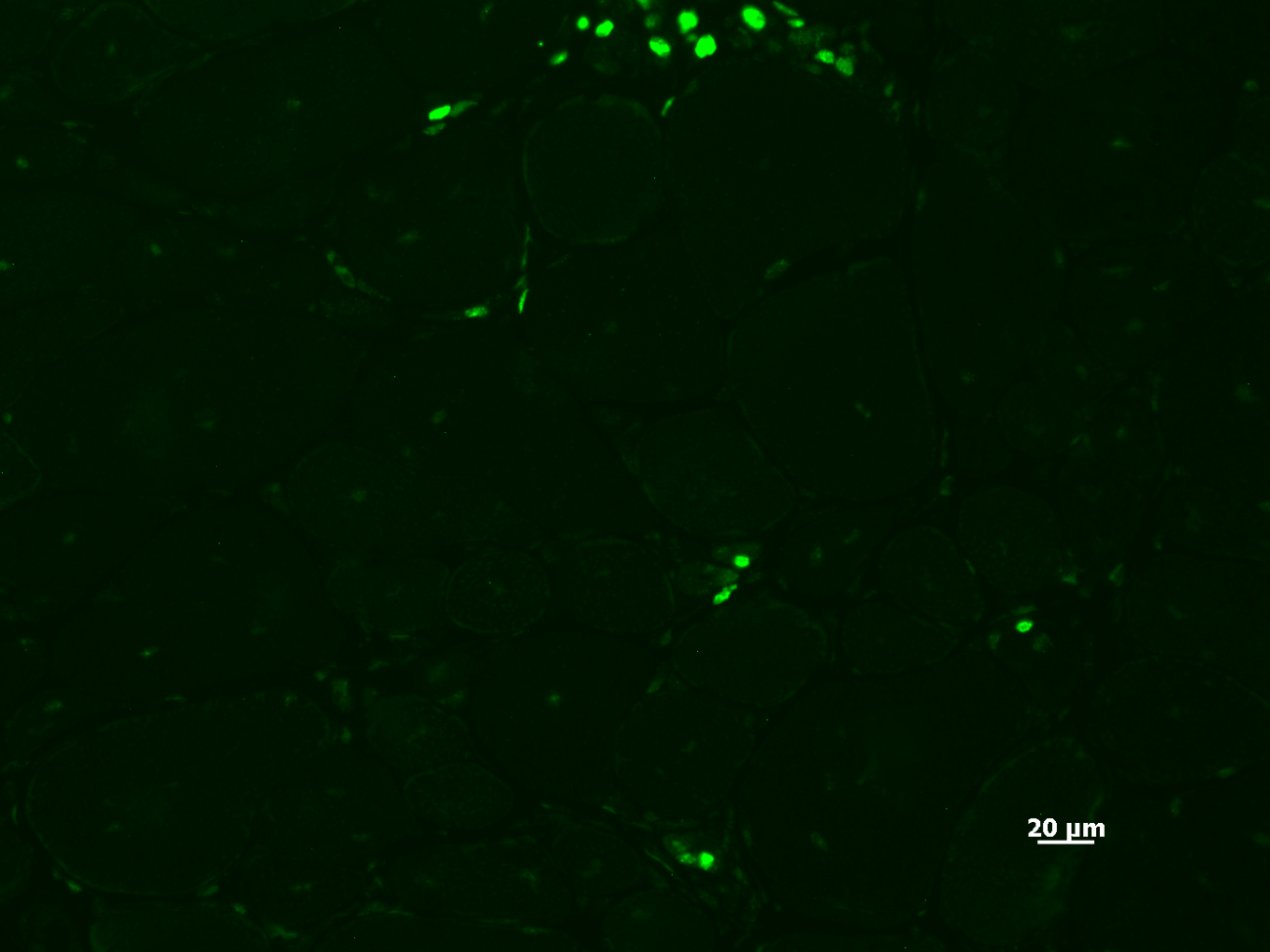


**Supplemental Figure 9.** Immunofluorescence of induced injury mouse Day 0 for dMyHC in (red), no labeling. Green labeling for Laminin and blue labeling for nuclei. Merged images. Magnification 100X.


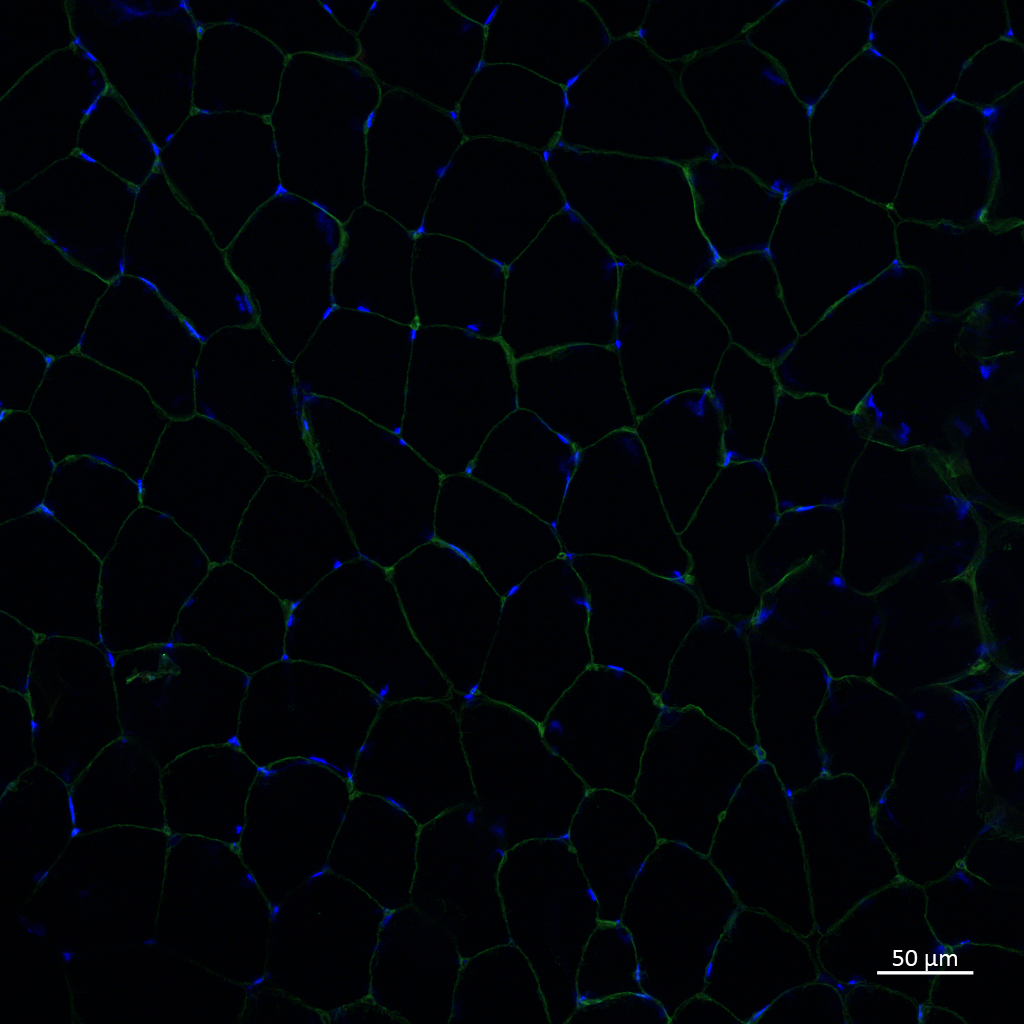


**Supplemental Figure 10.** Immunofluorescence of induced injury mouse after 5 days for dMyHC in (red). Green labeling for Laminin and blue labeling for nuclei (dapi). Merged images. Magnification 100X.


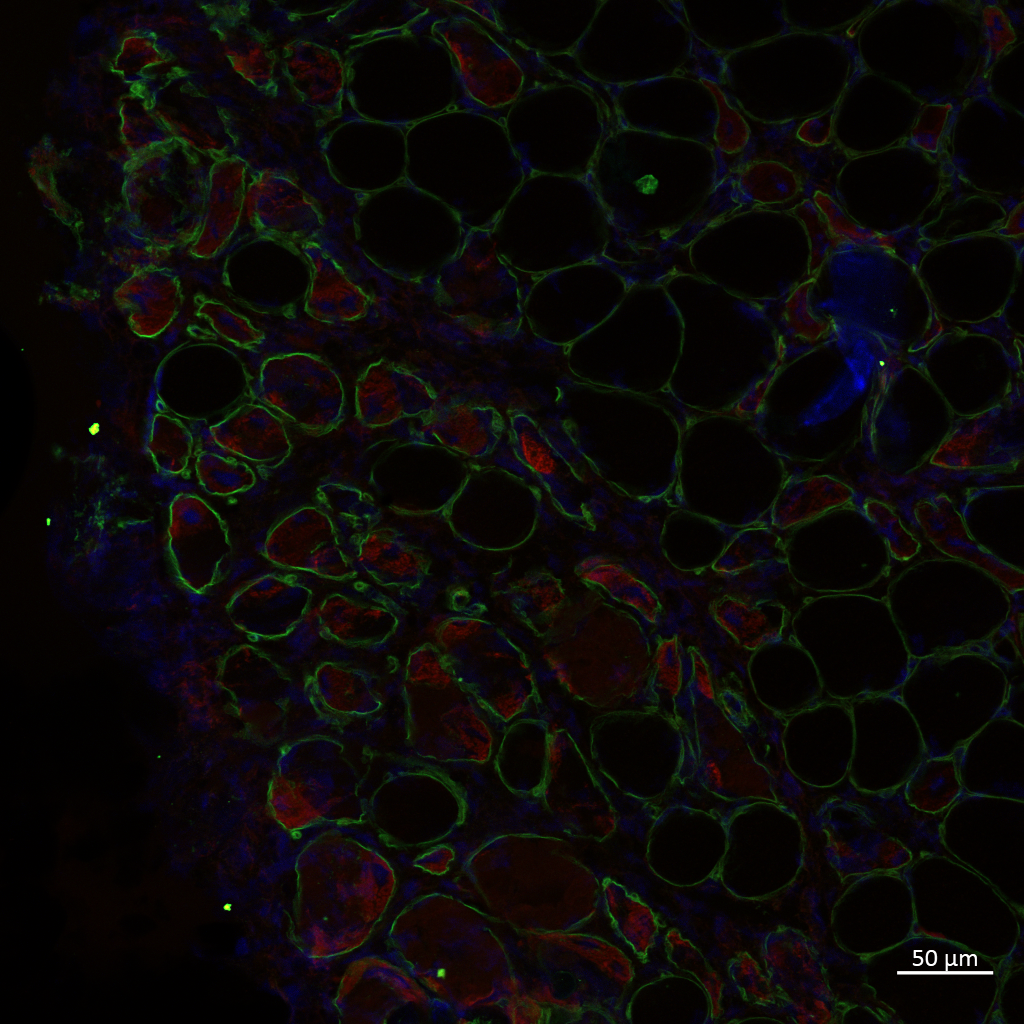


**Supplemental Figure 11.** Immunofluorescence of induced injury mouse after 60 days for dMyHC in (red), no labeling. Green labeling for Laminin and blue labeling for nuclei (dapi). Merged images. Magnification 200X.

**
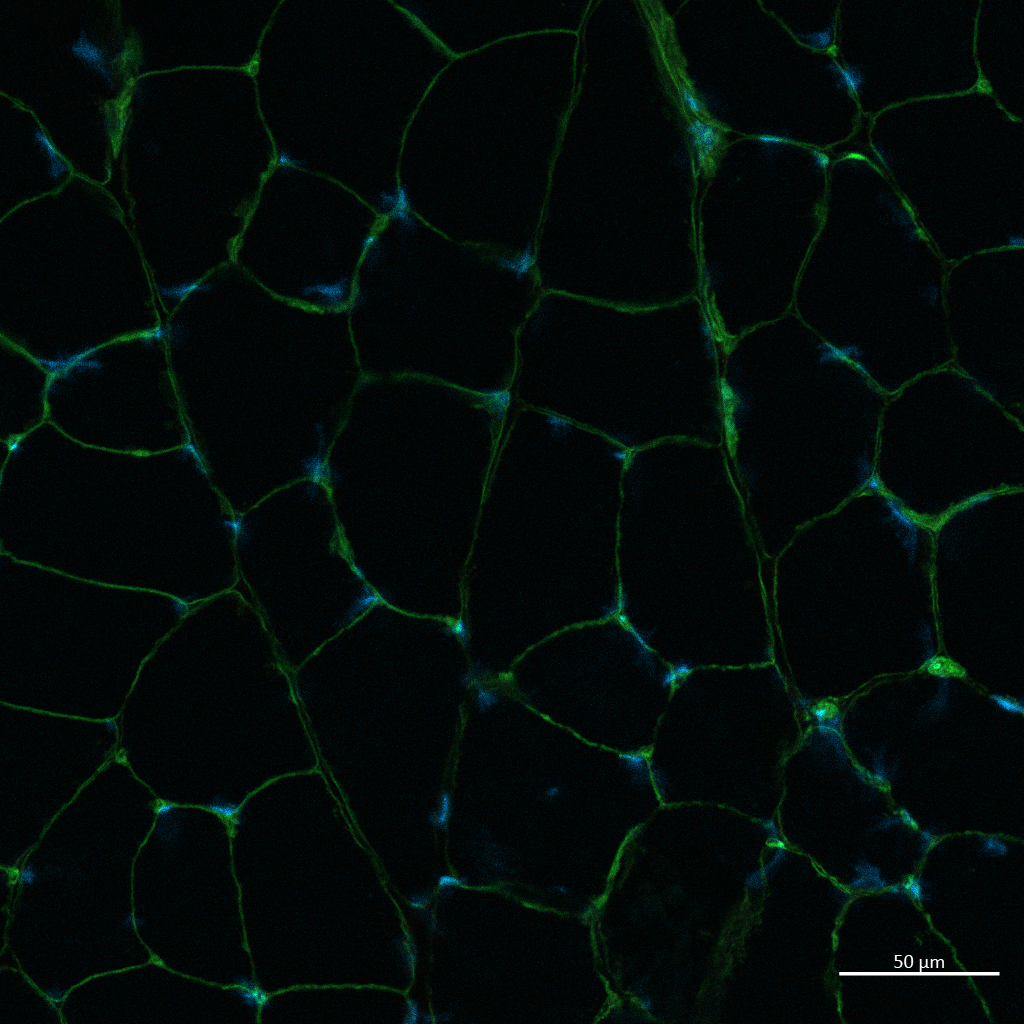
**

**Supplemental Figure 12.** Immunofluorescence of *Dmd^mdx^* mouse for dMHC (red). Green labeling for Laminin and blue labeling for nuclei. Merged images. Magnification 100X. Image capture conditions was standardized for the red labeling.


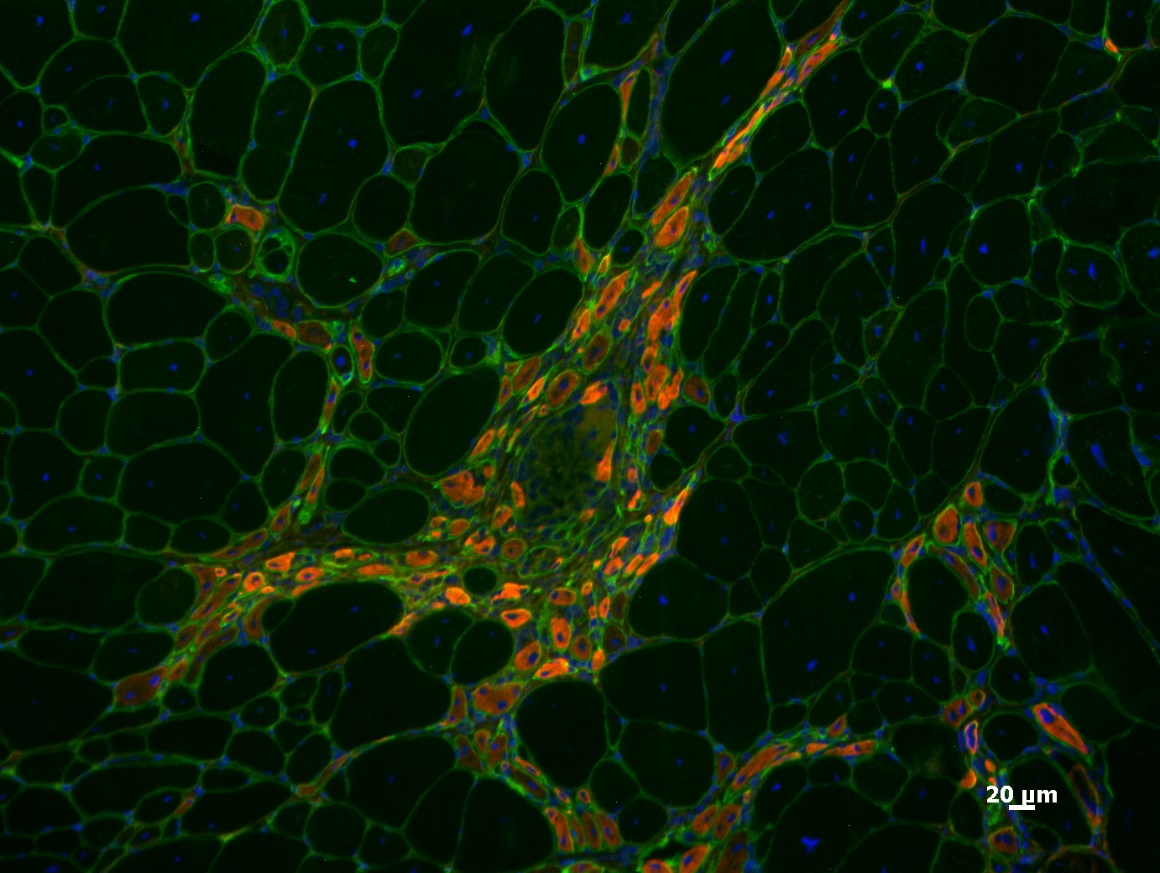


**Supplemental Figure 13.** Immunofluorescence of *Large^myd^* mouse for dMHC (red). Green labeling for Laminin and blue labeling for nuclei. Merged images. Magnification 100X.


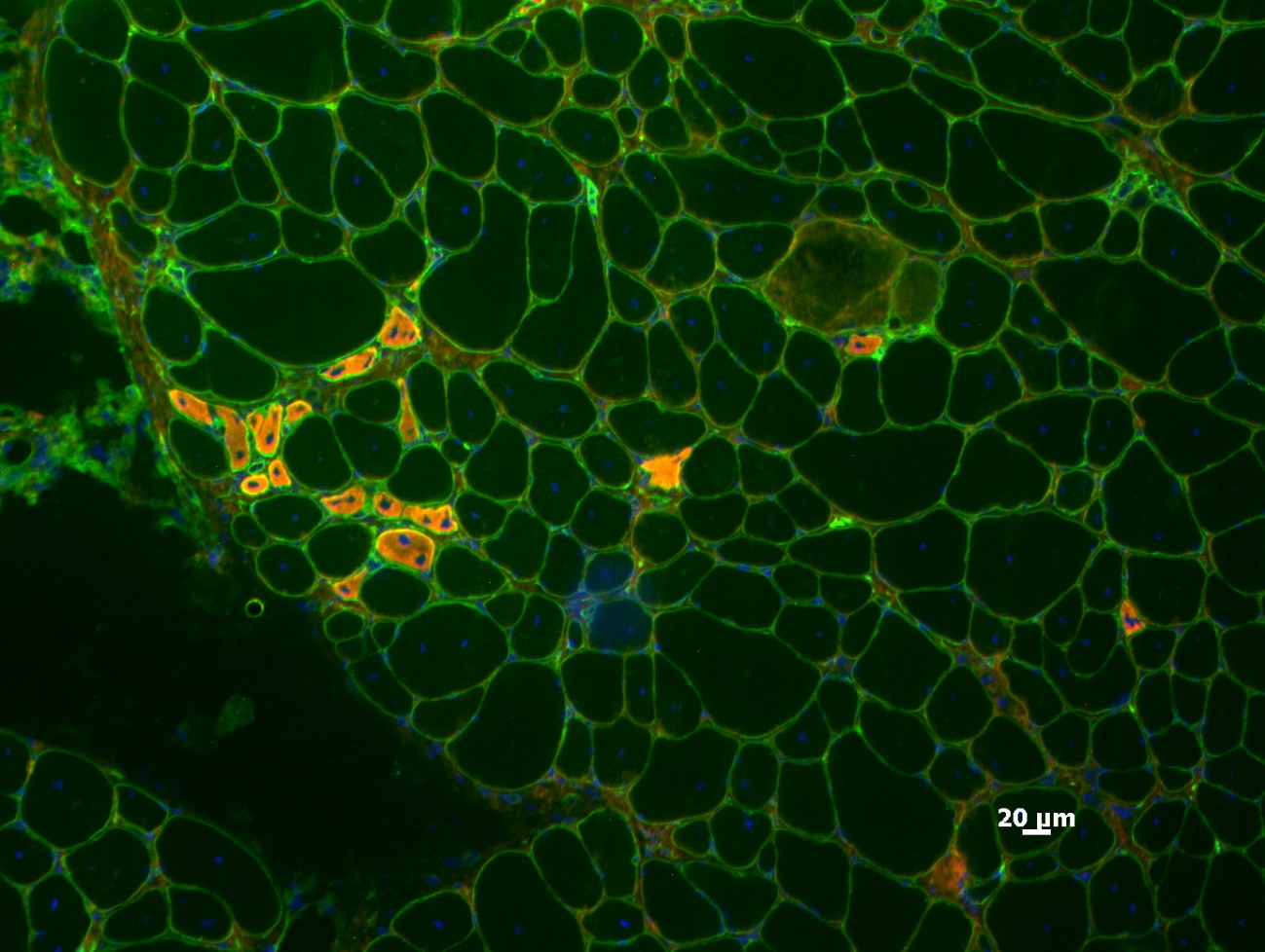


**Supplemental Figure 14.** Immunofluorescence of *Dmd^mdx^/Large^myd^* mouse for dMHC (red). Green labeling for Laminin and blue labeling for nuclei. Merged images. Magnification 100X.


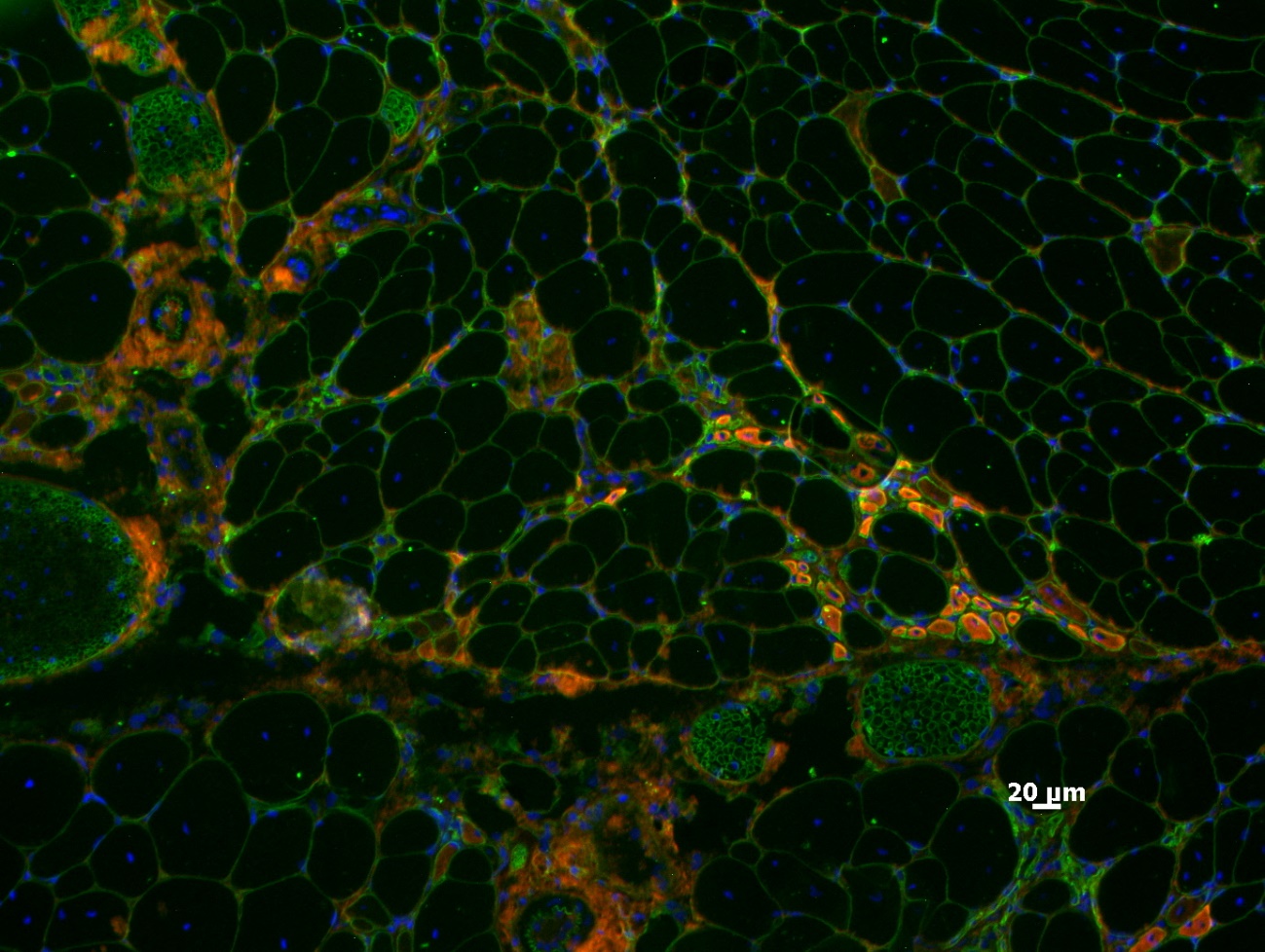


**Supplemental Figure 15.** Full image Picrosirius staining in WT mouse. Magnification 200X.


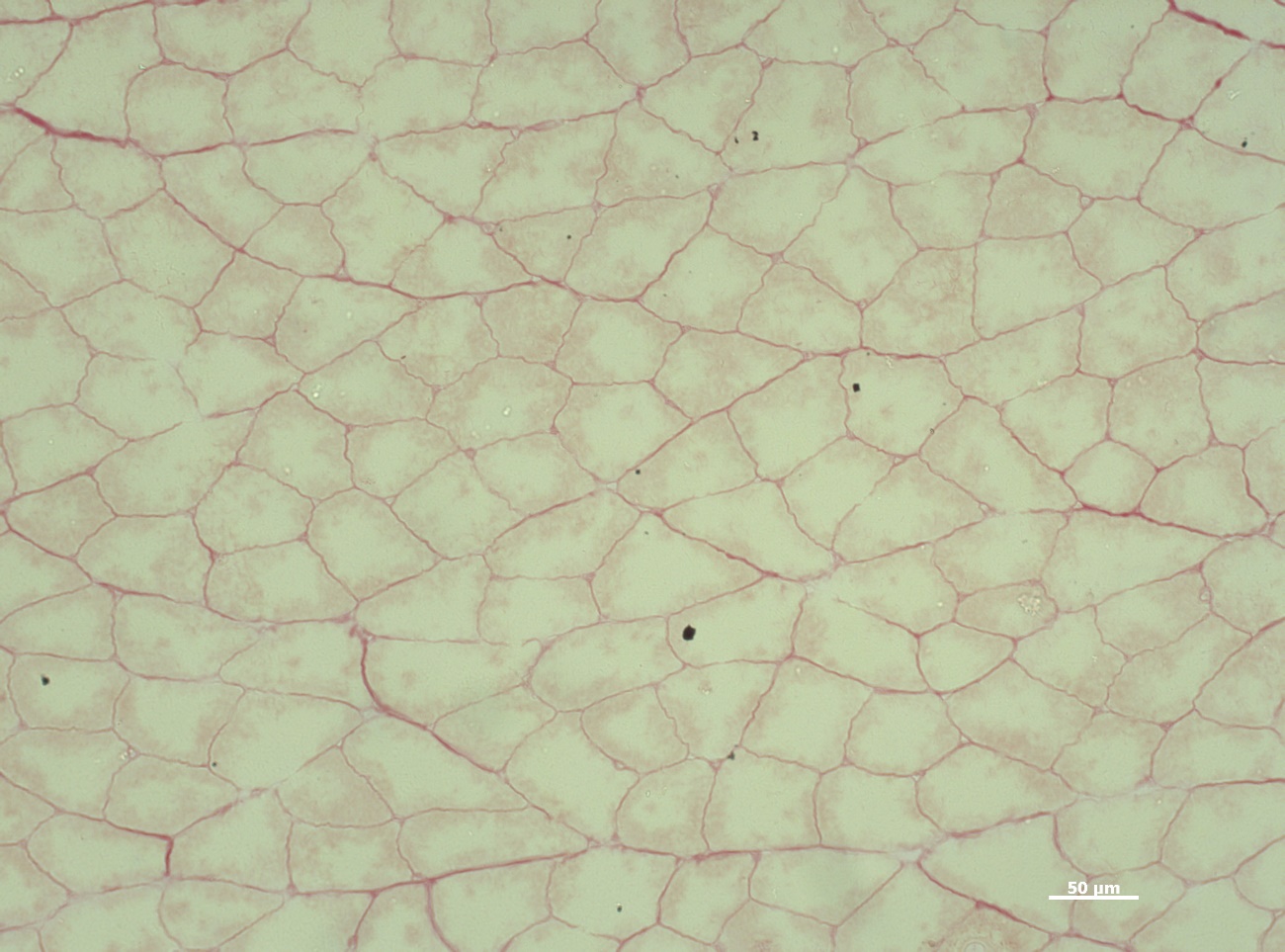


**Supplemental Figure 16**. Full image Picrosirius staining in *Dmd^mdx^* mouse. Magnification 200X.


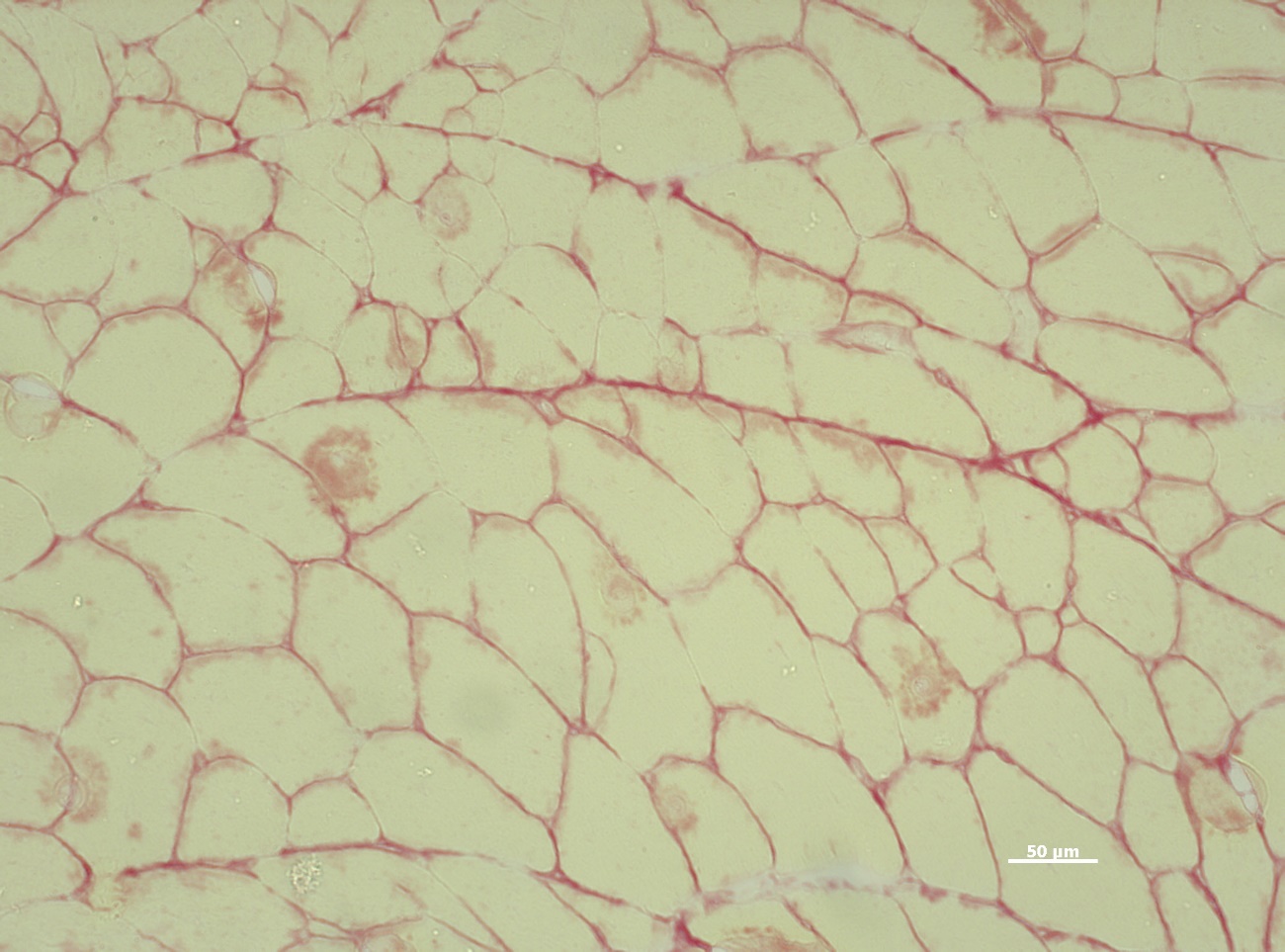


**Supplemental Figure 17**. Full image Picrosirius staining in *Large^myd^* mouse. Magnification 200X.


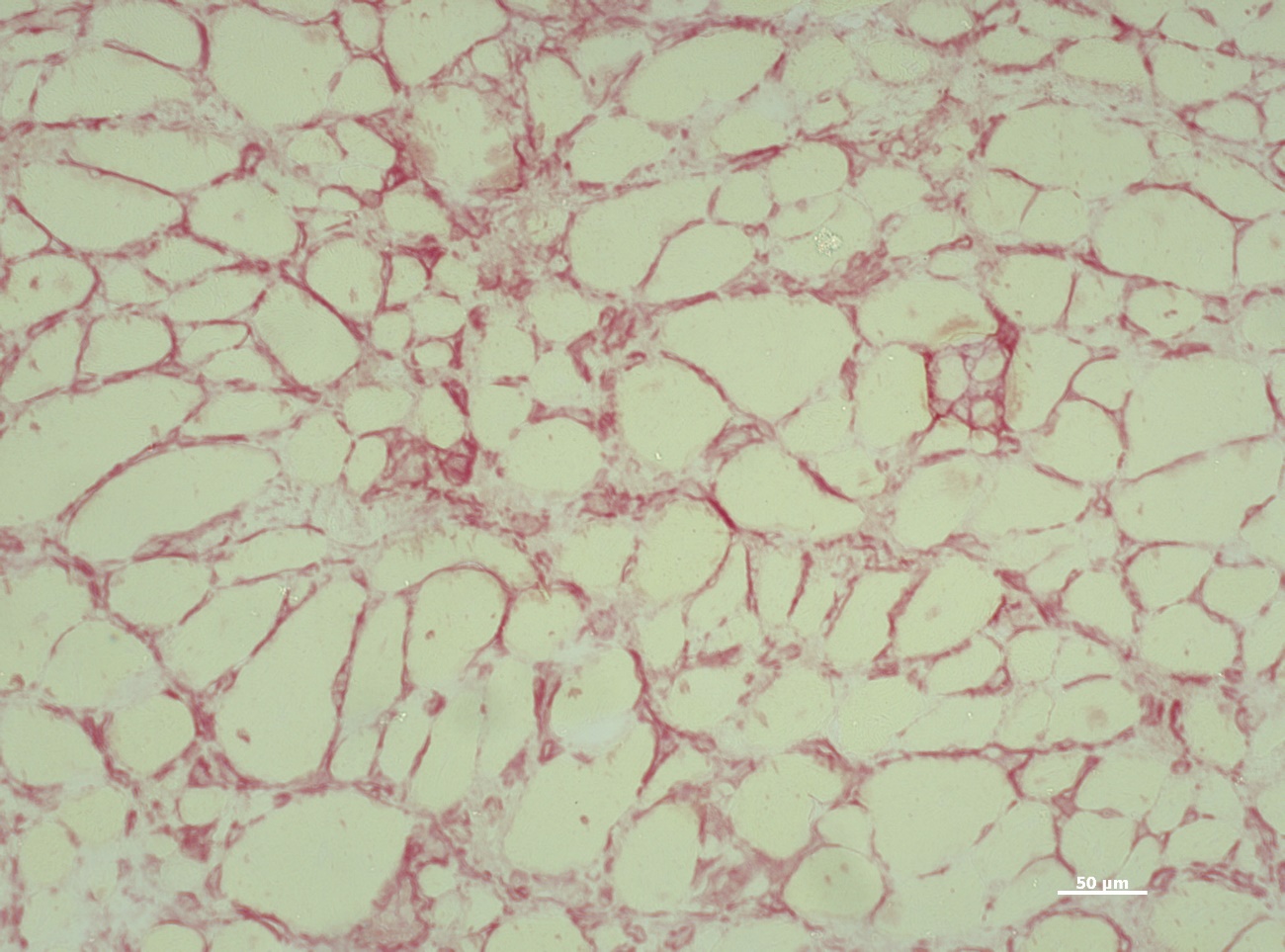


**Supplemental Figure 18**. Full image Picrosirius staining in *DMD^mdx^/Large^myd^* mouse. Magnification 200X.


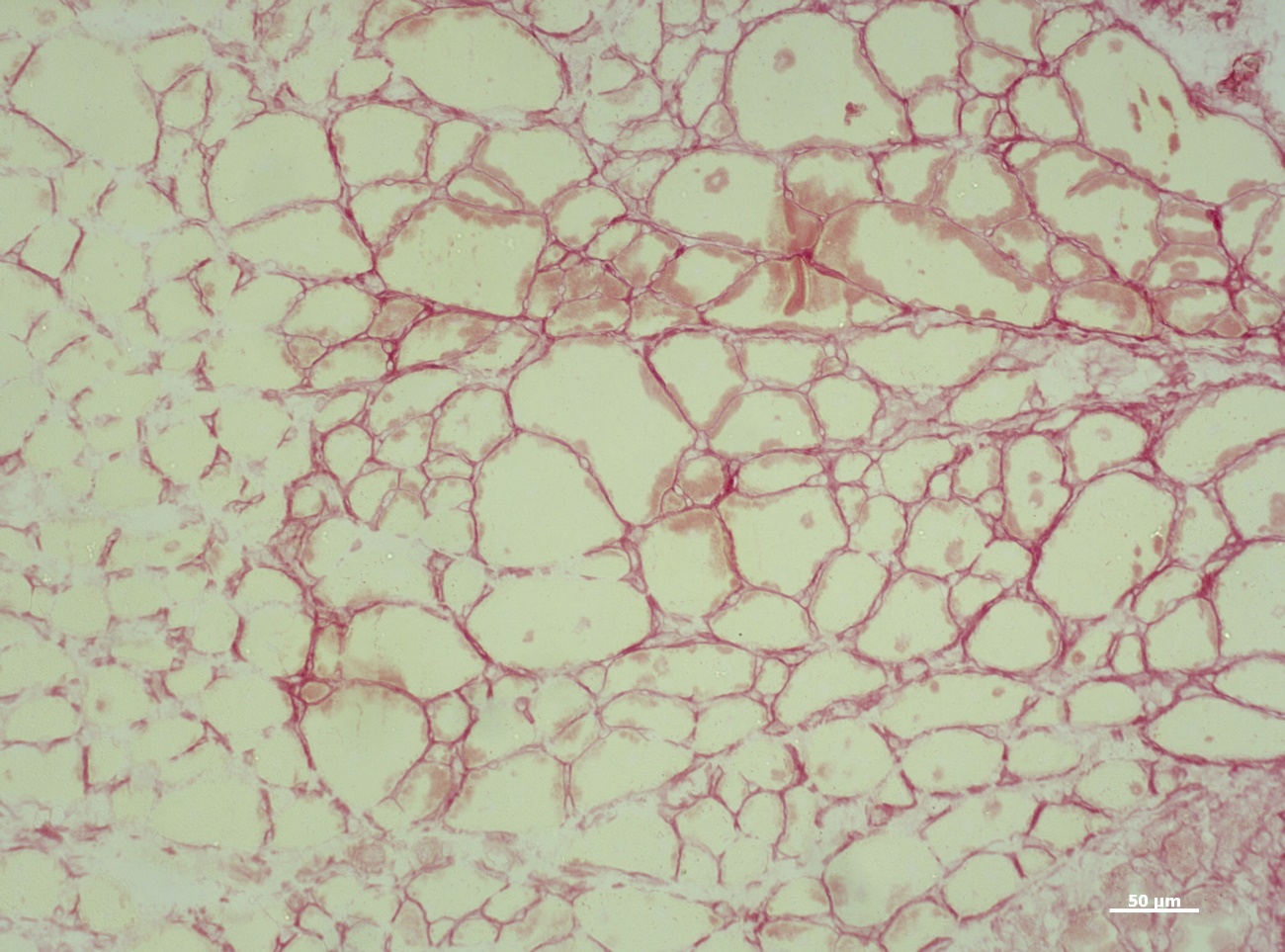


**Supplemental Figure 19.** Full image H&E staining in WT mouse. Magnification 100X.


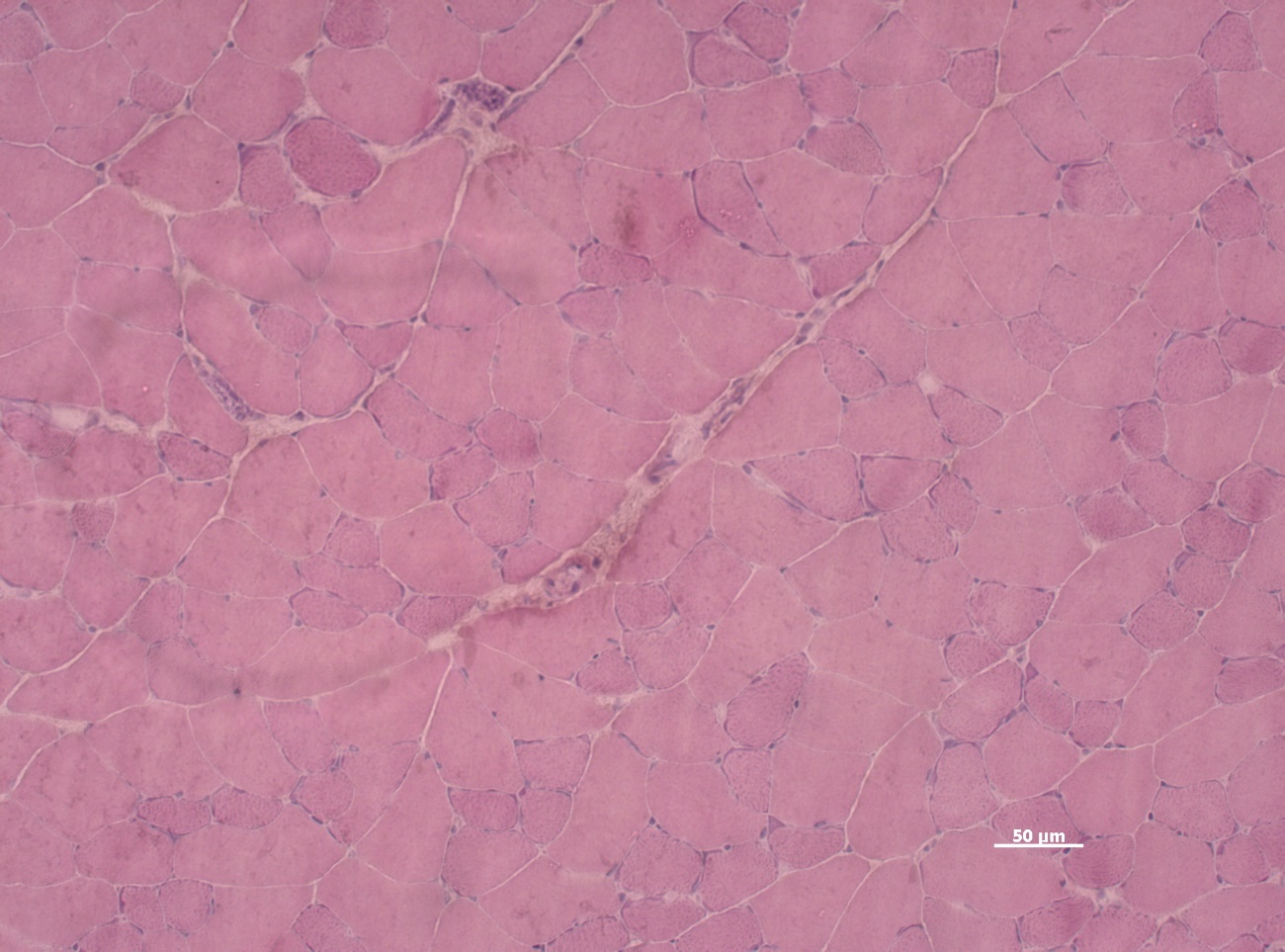


**Supplemental Figure 20.** Full image H&E staining in *Dmd^mdx^* mouse. Magnification 1000X.


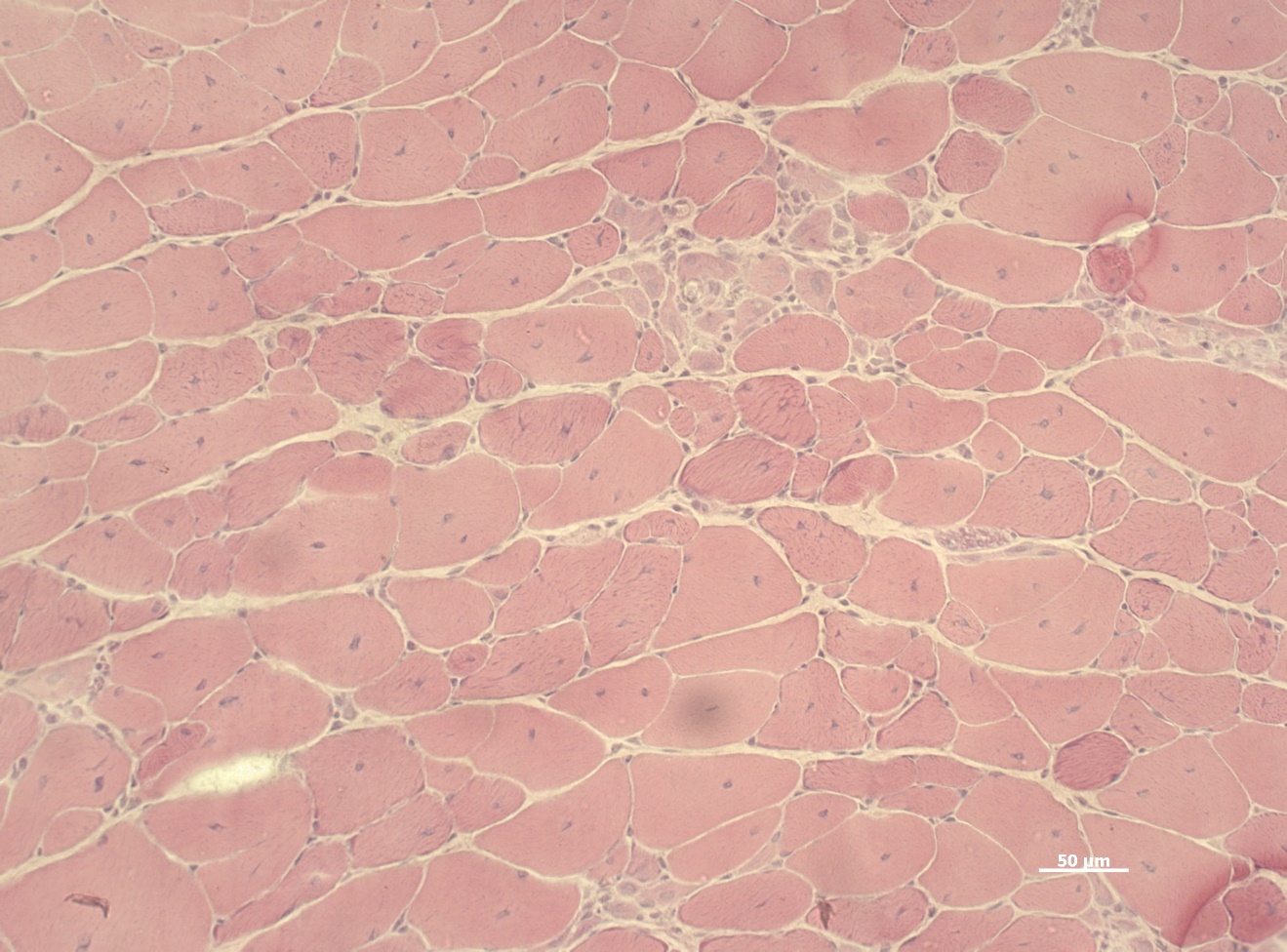


**Supplemental Figure 21.** Full image H&E staining in *Large^myd^* mouse. Magnification 100X.


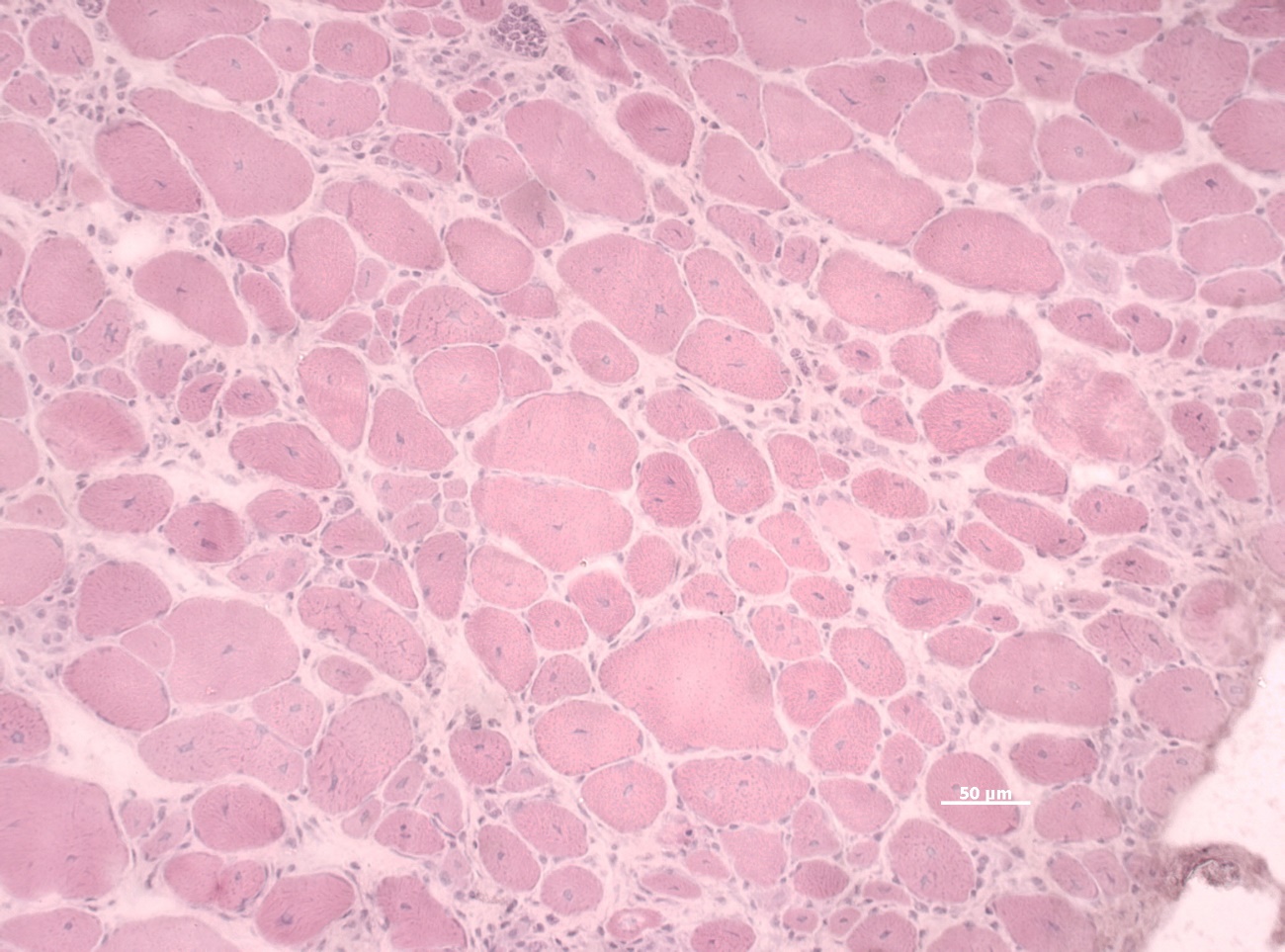


**Supplemental Figure 22.** Full image H&E staining in *Dmd^mdx^/Large^myd^* mouse. Magnification 100X.


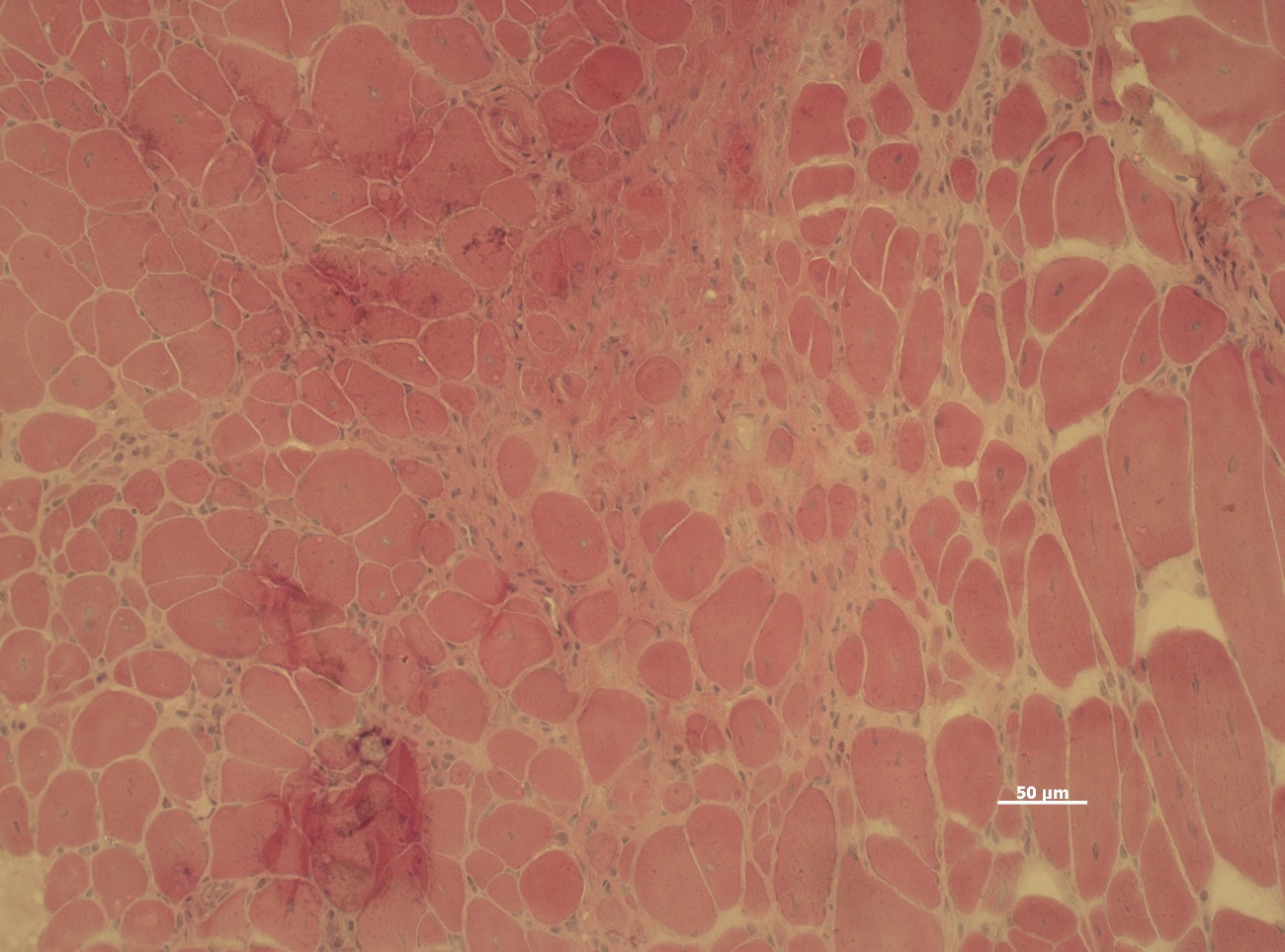


**Supplemental Figure 23.** This figure shows the original western blots that were made and are represented in Figure 4b, in which were used anti-MYOG and anti-GAPDH antibodies. Both membranes were stained with ponceau as an experimental control.

**
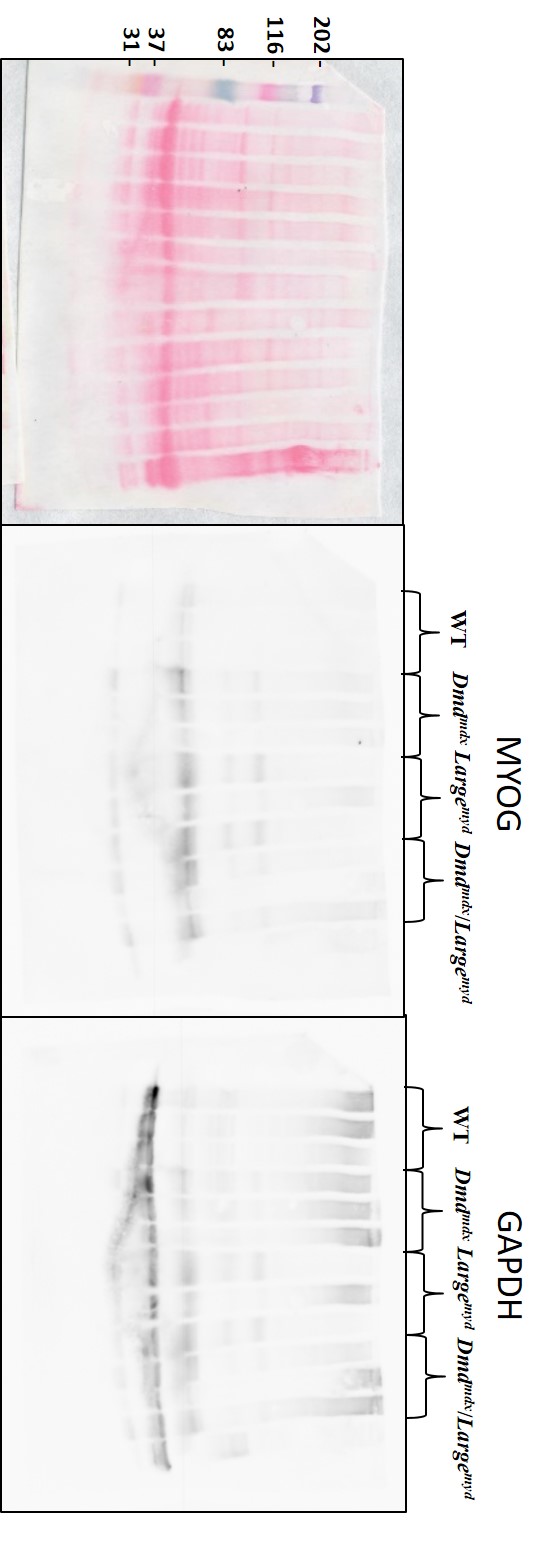

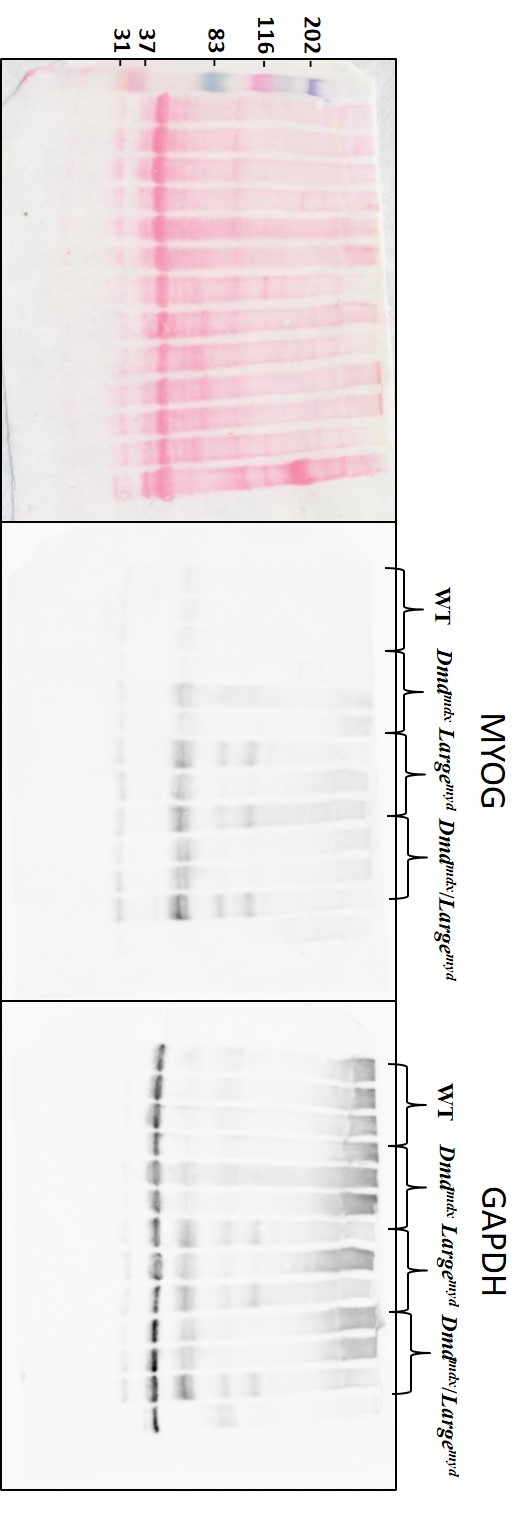
**

**Supplemental Figure 24.** This figure shows the original western blots that were made and are represented in Figure 1b, in which were used anti-PAX7 and anti-GAPDH antibodies. Both membranes were stained with ponceau as an experimental control.


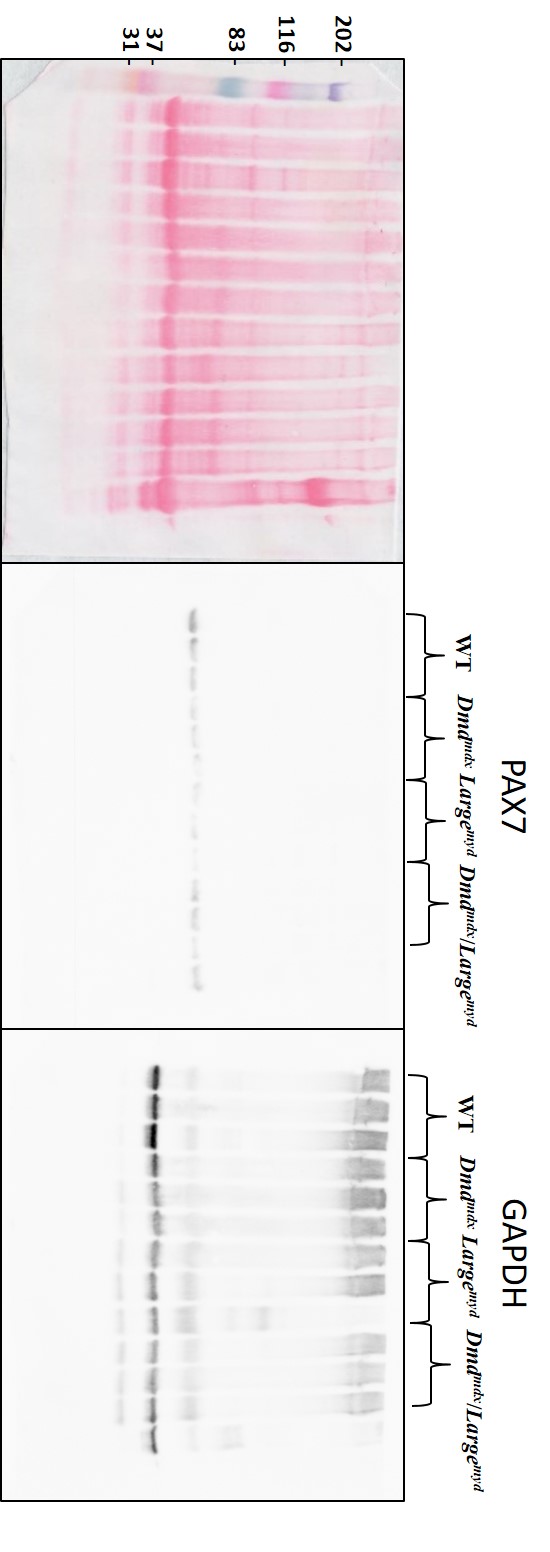

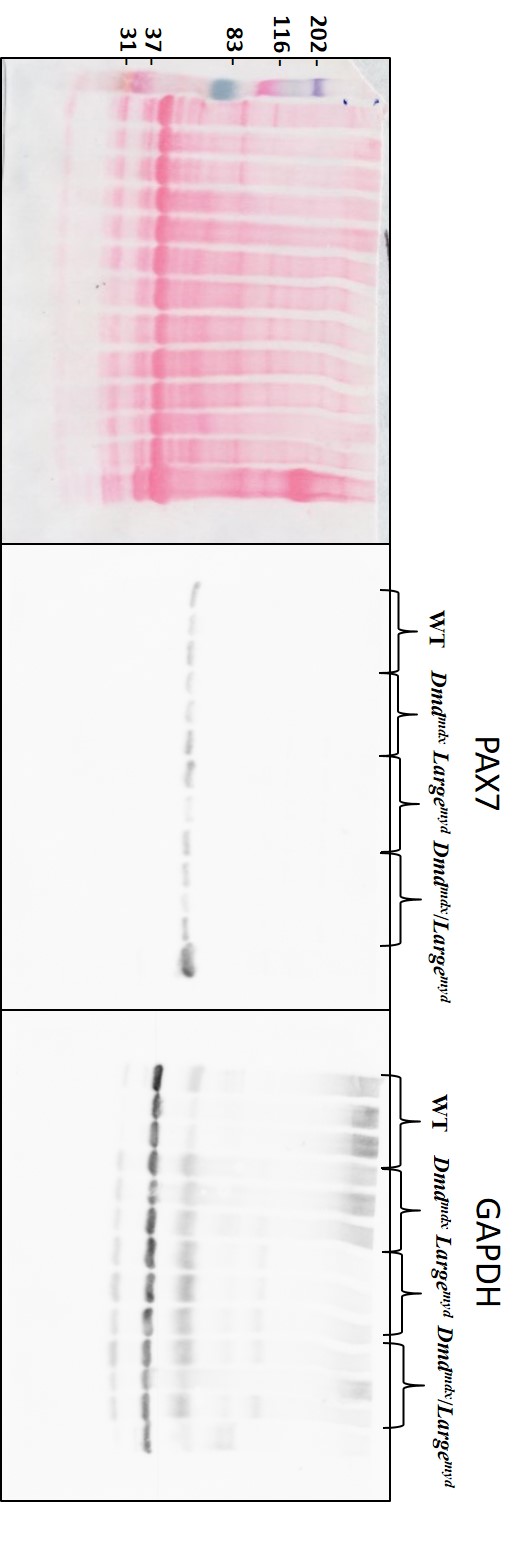

Supplement: Supplementary file 1 — Supplemental information [file 41598_2019_48156_MOESM1_ESM.docx]
